# Mammalian neurotoxins, *Blarina* paralytic peptides, cause hyperpolarization of human T-type Ca channel hCa_v_3.2 activation

**DOI:** 10.1016/j.jbc.2023.105066

**Published:** 2023-07-17

**Authors:** Yusuke Yano, Ryo Fukuoka, Andres D. Maturana, Satoshi D. Ohdachi, Masaki Kita

**Affiliations:** 1 Graduate School of Bioagricultural Sciences, Nagoya University, Nagoya, Japan; 2 Institute of Low Temperature Science, Hokkaido University, Sapporo, Japan

**Keywords:** mammalian venom, neurotoxin, peptide chemical synthesis, disulfide, calcium channel, molecular evolution, gating modifier toxin

## Abstract

Among the rare venomous mammals, the short-tailed shrew *Blarina brevicauda* has been suggested to produce potent neurotoxins in its saliva to effectively capture prey. Several kallikrein-like lethal proteases have been identified, but the active substances of *B. brevicauda* remained unclear. Here, we report *Blarina* paralytic peptides (BPPs) 1 and 2 isolated from its submaxillary glands. Synthetic BPP2 showed mealworm paralysis and a hyperpolarization shift (−11 mV) of a human T-type Ca^2+^ channel (hCa_v_3.2) activation. The amino acid sequences of BPPs were similar to those of synenkephalins, which are precursors of brain opioid peptide hormones that are highly conserved among mammals. However, BPPs rather resembled centipede neurotoxic peptides SLPTXs in terms of disulfide bond connectivity and stereostructure. Our results suggested that the neurotoxin BPPs were the result of convergent evolution as homologs of nontoxic endogenous peptides that are widely conserved in mammals. This finding is of great interest from the viewpoint of the chemical evolution of vertebrate venoms.

Venoms of mammalian origin are rare, and their mechanisms of action, chemical evolution, and relationships with their producers remain poorly understood. Several plesiomorphic mammals, such as platypus (1), solenodon (2), and shrews (3, 4, 5), have been suggested to possess venoms for self-defence or predation. Recent toxicogenomic and molecular phylogenetic analyses have unveiled the diversification history of venomous mammals (6, 7). For example, the family Solenodontidae diverged from other Eulipotyphla in the Paleocene, and the Cuban solenodon (*Solenodon cubanus*) diverged from the Hispaniolan venomous species (*S. paradoxus*) in the Early Pliocene (8, 9, 10). As in this study, natural venoms have been mainly investigated by genomics and proteomics approaches (11, 12, 13, 14), and few studies have focused on compounds purified by chemical approaches. Still, several mammalian toxic substances have been isolated and characterized to date, including those from platypus (15, 16) and shrews (17, 18, 19, 20, 21, 22).

While most of the shrew species worldwide are not venomous, a few of them in the genera *Neomys* and *Blarina* produce toxic substances in their saliva. It has recently been demonstrated that venom from the Eurasian water shrew *Neomys fodiens* exhibits paralytic and cardioinhibitory activity against beetles and frogs (17) and decreases the conduction velocity of sciatic nerve and the force of calf muscle contraction in frogs (18). In addition, the venoms of *N. fodiens* and the common shrew *Sorex araneus* were shown to exhibit weak hemolytic activity (19). On the other hand, the short-tailed shrew *Blairna brevicauda* is a relatively small (7 ∼ 10 g in body weight) mammal that feeds on both vertebrates (murid rodents and frogs) and invertebrates (insects and earthworms) (23, 24, 25). The *Blarina* shrew has been suggested to utilize potent venoms to effectively capture a variety of preys (3). Several kinds of glycosylated kallikrein-like proteases, blarina toxin (BLTX) (20) and blarinasins (21, 22) have been isolated from this species. Despite the high similarity in amino acid sequence to human and other mammalian kallikreins, BLTX showed lethal toxicity against mice. Meanwhile, both BLTX and blarinasin showed no paralytic effects on invertebrates, so we thought that *Blarina* shrews might have some other neurotoxic substance(s) in their saliva. As a result, two novel peptides were isolated, *Blarina* paralytic peptides (BPPs) 1 and 2. In this report, we describe the structure, chemical synthesis, and biological activity of BPPs.

## Results

### Isolation of BPPs and their amino acid sequences

To evaluate paralytic neurotoxins, we extracted the submaxillary glands of *B. brevicauda* with saline (7 ∼ 10 g body weight, captured in Michigan, USA, in 2002 to 2003 (20); the substances in tissues were stable in acetone at −30 °C for more than 20 years!). As demonstrated by Stewart *et al.* (26), approximately 1 in 70 individual samples of this extract reliably induced paralysis and convulsions in mealworms (larva of *Zophobas atratus*, 0.7 ∼ 1.0 g body weight) immediately after *i.p.* administration. This effect was not inhibited by aprotinin, suggesting the presence of lower-molecular-weight neurotoxins different from BLTX and other serine proteases.

Extraction and purification of BPPs from submaxillary glands were performed in 2002, 2007, 2013, and 2020. Mealworm paralytic activity (1/70 individuals) was reproducibly observed in all four experiments. In addition to BPPs, the SDS-PAGE pattern of crude venom extracts performed in 2013 (Fig. S1) was highly similar to those in 2002 (21). Thus, we concluded that the main protein components including BLTX as well as BPPs were stable for a long time.

**Figure 1:**
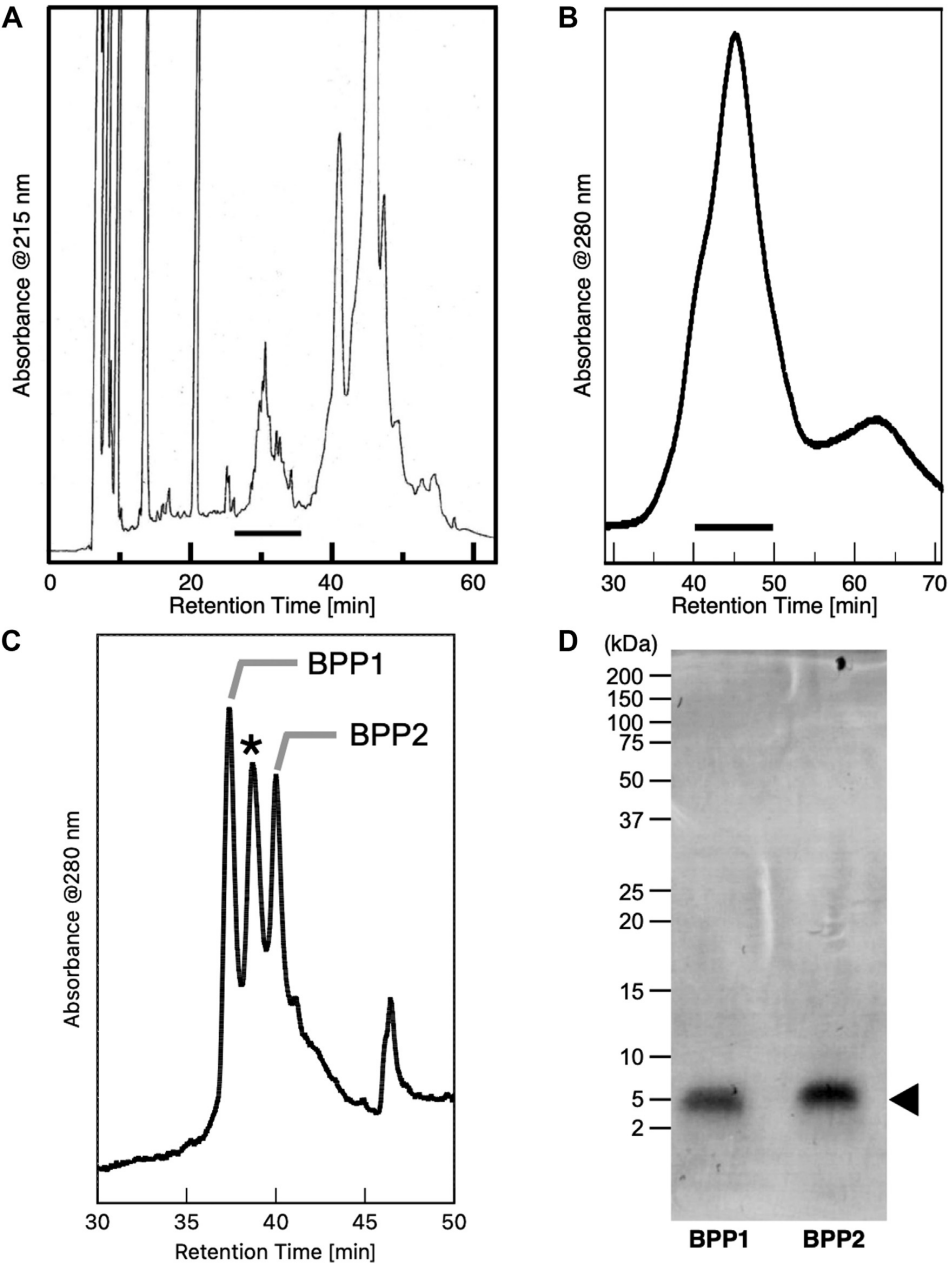
**Purification of BPPs 1 and 2.***A*, RP-HPLC analysis of the *Blarina* submaxillary gland extract (1/10 individual) using a wide-pore (300 Å) C_4_ column. The solid bar fractions (*t*_R_: 23 ∼ 36 min) were active. *B*, gel-permeation HPLC. The solid bar fractions (*t*_R_: 40 ∼ 50 min) were active. *C*, RP-HPLC using a C_30_ column with an aq. MeCN–HFBA solvent system. Two peaks were isolated as BPPs 1 and 2 (*t*_R_ = 37.4 and 40.0 min, respectively). An *asterisk* means BPP analogs (*t*_R_ = 38.7 min) with molecular weights similar to those of BPPs. *D*, SDS-PAGE analysis of purified BPPs. An arrowhead means single bands of BPPs 1 and 2 with approximately 5.2 and 5.5 kDa, respectively.

Purification of BPPs was accomplished by three-step column chromatography as follows. First, the saline extracts were separated by a reversed-phase (RP)-HPLC using a wide-pore (300 Å) C_4_ column (Fig. 1*A*). Only the fraction with a retention time of 26∼36 min showed mealworm paralytic activity, while the major higher-molecular-weight proteinaceous substances including BLTX and blarinasin were separately eluted at 43∼48 min (Fig. S1, *A* and *B*). The active fractions were next subjected to a gel-permeation HPLC (Fig. 1*B*) to give an active fraction as a major peak (43∼47 min), which mainly contained peptides with molecular weights of 5∼6 kDa. Final purification by a RP-HPLC using a C_30_ column with an aq. MeCN–heptafluorobutyric acid (HFBA) solvent system (Fig. 1*C*) afforded three major constituents, two of which, BPPs 1 and 2 (*t*_R_: 37.4 and 40.0 min), were isolated as single compounds.

**Figure 2:**
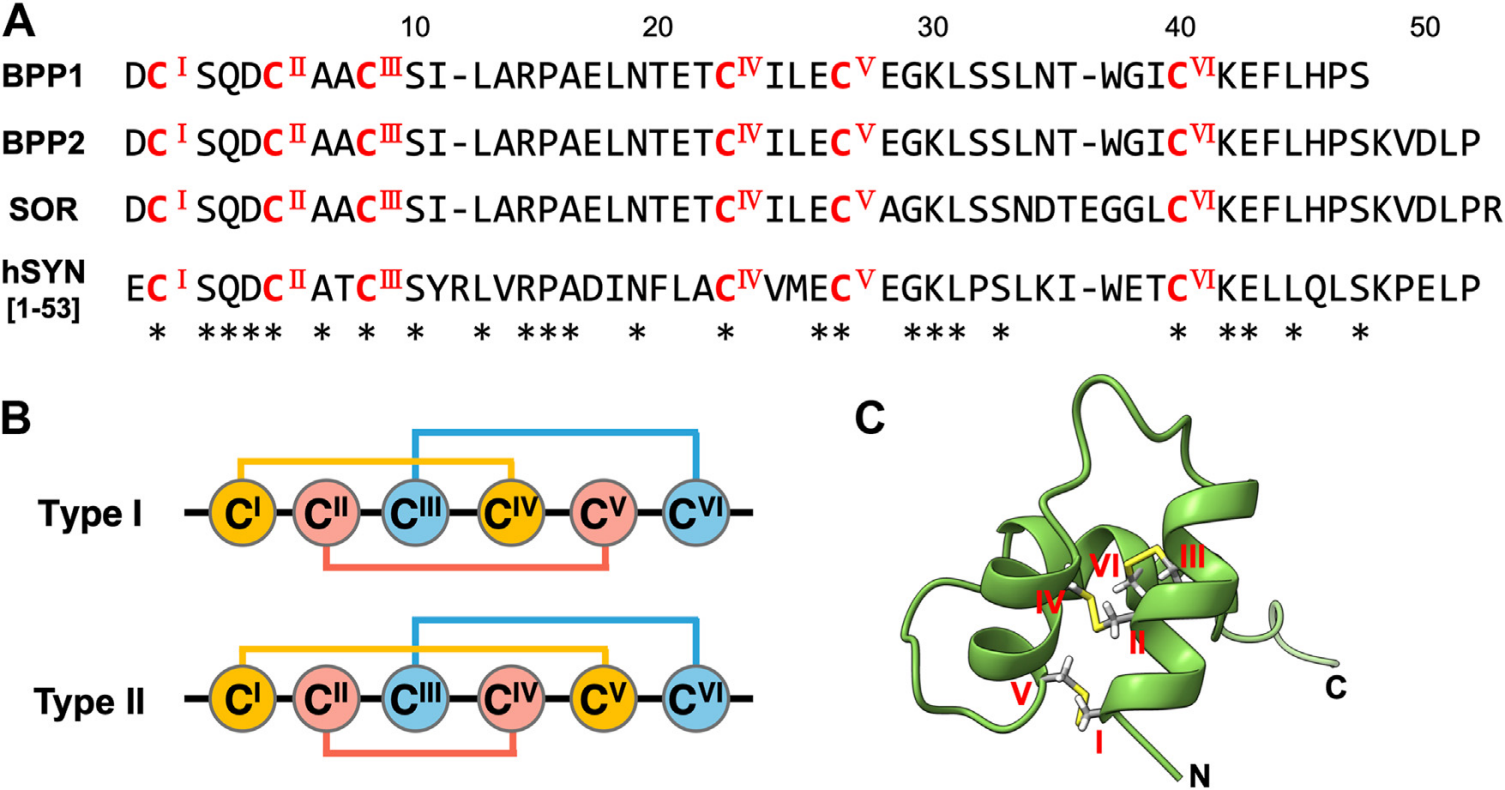
**Structures of BPPs.***A*, the primary amino acid sequences of BPP1, BPP2, soricidin (SOR), and the N-terminal part of human synenkephalin (hSYN [1–53]). Amino acid position numbers correspond to those of BPPs. *Asterisks* indicate conserved residues among the four peptides. *B*, schematic representations of the two proposed disulfide bond connectivity of BPPs. *C*, proposed stereostructure of BPP2 (Type-II SS connectivity) obtained by ColabFold.

In SDS-PAGE analysis, BPPs 1 and 2 were both detected as single bands of approximately 5.2 and 5.5 kDa, respectively (Fig. 1*D*). The primary amino acid sequences of BPPs were initially analyzed by MALDI-TOF MS and MS/MS analysis of the samples treated with DTT (reduction), iodoacetamide (IAM) (alkylation), and subsequent digestion with trypsin or glutamyl endopeptidase (Glu-C) (Figs. S2 and S3). In addition, the sequences of BPPs including Ile/Leu and Lys/Gln discrimination were established by comparison with the transcriptome data of submaxillary glands (EMBL-EBI, Sequence ID: MT559766) (12). As a result, BPPs had sequences similar to the precursor part of proenkephalin (called as synenkephalin), which includes an opioid peptide hormone expressed in mammalian brain. We established that BPPs 1 and 2 had single peptide chains of 47 and 52 amino acid residues, respectively, with common N-terminal sequences (Fig. 2*A*). These sequences highly resembled that of soricidin (SOR, 54 amino acid residues, 5.8 kDa), a paralytic peptide isolated from *B. brevicauda* by Bowen *et al.* (27), but differed in six C-terminal region residues (N^34^–R^53^). We carefully checked whether SOR contained in our samples separated by gel-permeation and RP-HPLC, but was not detected. It was recently reported that proenkephalins are contained in the submaxillary glands of *N. fodiens* and *S. araneus*, but their structures and functions have not been established (19).

### Prediction of disulfide bond connectivity

MALDI-MS analysis revealed that BPPs 1 and 2 both had three intramolecular disulfide bonds *via* the six Cys residues. The D^1^–K^41^ regions of BPPs were highly resistant to digestive enzymes such as trypsin or Glu-C without reductive *S*-alkylation of Cys residues. Thus, we conjectured that these disulfide bonds may highly stabilize the folded structures of BPPs. A BLAST search (28) revealed that the amino acid sequences of BPPs were highly similar to that of human synenkephalin (29, 30) (hSYN [1–53], 59.6% identity) and to those of other mammals. The apparent pI values of BPPs 1 and 2 were 4.49 and 4.55, respectively, which are slightly less than that of hSYN [1–53] (4.86). The disulfide bond connectivity of recombinant rat synenkephalin [1–73] (with an extended C-terminal sequence) was shown to be Cys(I)–Cys(IV), Cys(II)–Cys(V), and Cys(III)–Cys(VI) (called as Type I) (Fig. 2*B*) (31). On the other hand, we used a stereostructure prediction program based on the amino acid sequences, ColabFold (32) (a Google Colab version of AlphaFold2 (33) using MMSeq2 (34)). This analysis proposed that BPPs 1 and 2 had another disulfide bond connectivity, Cys(I)–Cys(V), Cys(II)–Cys(IV), and Cys(III)–Cys(VI) (called as Type II) (Figs. 2, *B* and *C* and S4). Due to the limited amounts of natural BPPs, it was not possible to determine which disulfide bond pattern they have, so we attempted to synthesize BPP2 and evaluate its structure and biological activity.

### Synthesis of BPP2

**Figure 3:**
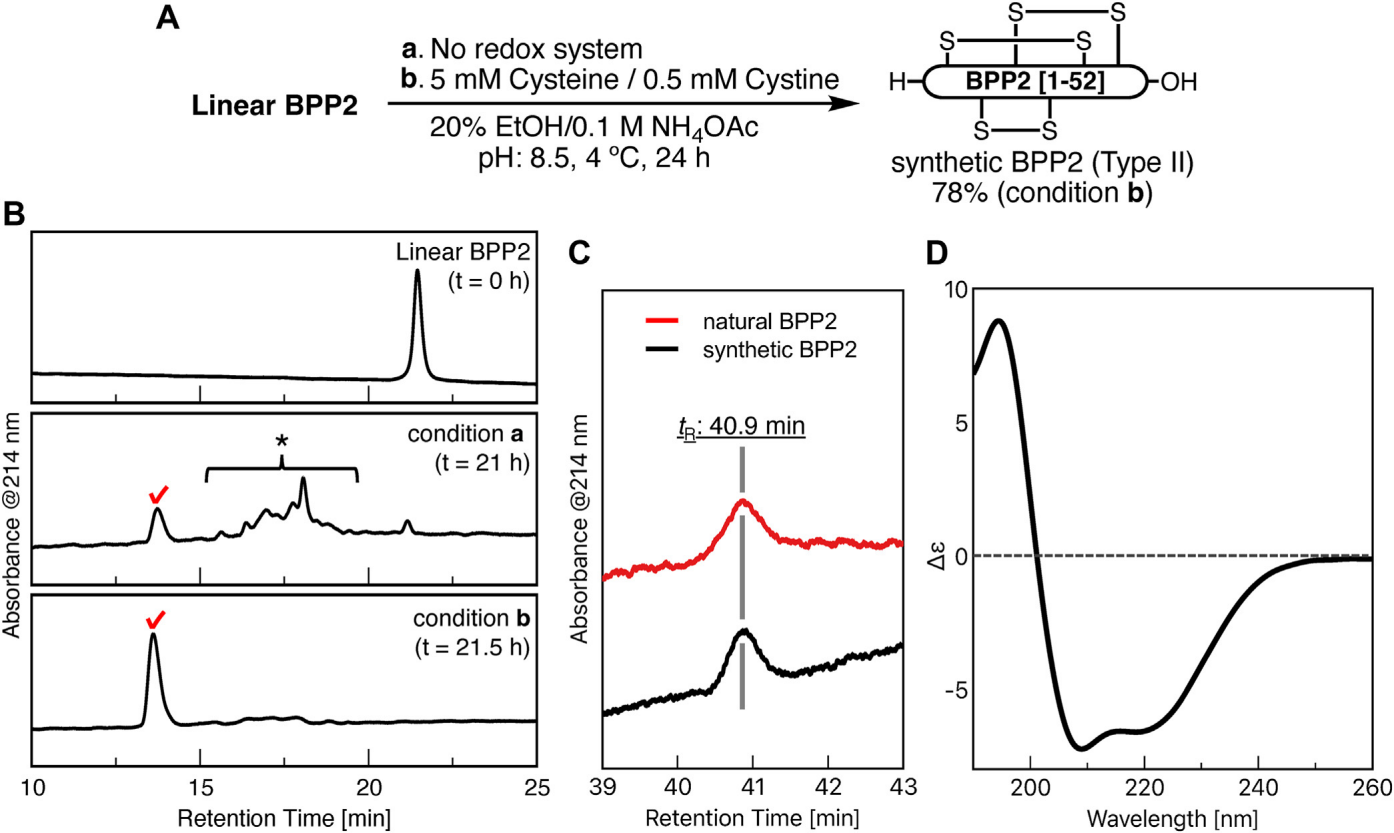
**Synthesis and identification with natural BPP2.***A*, preparation of BPP2 using redox conditions. *B*, analysis of the refolded products. HPLC conditions: Develosil ODS HG-5 (5 μm, ϕ 4.6 mm × 250 mm), 25% to 55% aq. MeCN/0.1% TFA gradient for 30 min, flow: 1.0 ml/min, 23 °C, monitoring at UV 214 nm. A check mark represents synthetic BPP2 (*t*_R_ = 13.6 min). An *asterisk* includes misfolded BPP2 analogs (*t*_R_ = 16 ∼ 19 min). *C*, comparison of the retention times between natural and synthetic BPP2. HPLC conditions: Develosil RP-AQUEOUS-AR-5 (5 μm, ϕ 4.6 mm × 250 mm), 20% to 50% aq. MeCN/H_2_O (containing 0.054%–0.060% HFBA) linear gradient for 60 min, flow: 0.5 ml/min, 23 °C, monitoring at UV 214 nm. *D*, CD spectrum of synthetic BPP2 (20 μM) in 50 mM potassium phosphate (pH 7.0) at 25 °C.

We planned to synthesize BPP2 from the linear peptide [1–52] *via* spontaneous disulfide bond formation (Fig. 3*A*). The N-terminal thioester [1–22] and the C-terminal cysteine [23–52] segments were prepared by a conventional Fmoc-solid-phase peptide synthesis method. Native chemical ligation (35) of these two segments afforded a linear peptide [1–52] in 37% yield, in which 4-mercaptophenylacetic acid and acetylacetone were used for thioesterification, and tris(2-carboxyethyl)phosphine hydrochloride (TCEP·HCl) was used for reducing agent (36). Finally, disulfide bond formation of linear peptide [1–52] with 5 mM cysteine/0.5 mM cystine in 20% aq. EtOH and 0.1 M NH_4_OAc (37) afforded BPP2 in 78% yield, which was detected as an almost single peak by HPLC analysis (Fig. 3*B*, *t*_R_ = 13.6 min). Both the redox reagents and alcoholic solvents were essential to obtain BPP2 with high convergence, otherwise several misfolded analogs with different disulfide bond patterns mainly formed (*t*_R_ = 16∼19 min). Since these misfolded analogs were successfully converted into BPP2 under the same refolding conditions as above, synthetic BPP2 was found to be the most thermodynamically stable product. Finally, both of the HPLC retention times and the MALDI MS data of synthetic BPP2 were identical to those of natural BPP2 (Fig. 3*C*).

### Disulfide bond connectivity analysis of synthetic BPP2

**Figure 4:**
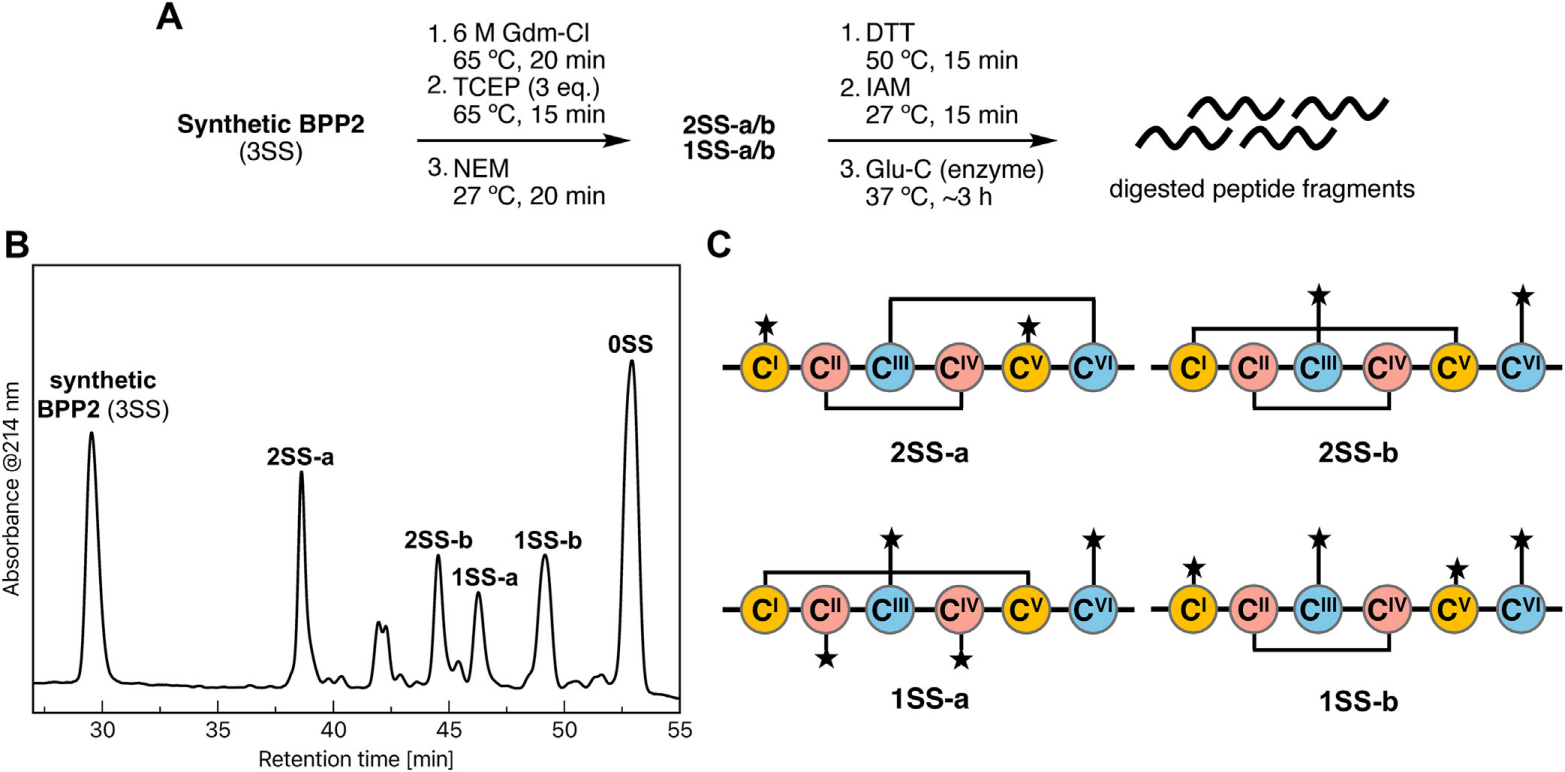
**Disulfide bond connectivity of BPP2.***A*, overview of the degradation scheme of BPP2 (called 3SS). *B*, HPLC analysis of the partially reduced peptides. HPLC conditions: Develosil ODS HG-5 (5 μm, ϕ 4.6 mm × 250 mm), 20% aq. MeCN/0.1% TFA, 5 min; 20% to 50% aq. MeCN/0.1% TFA linear gradient for 60 min, flow: 1.0 ml/min, 25 °C, monitoring at UV 214 nm. *C*, Structures of 2SS-a/b and 1SS-a/b. ★ Means the alkylated site with NEM. 0SS Means the fully NEM-alkylated peptide.

To determine the disulfide bond connectivity of synthetic BPP2, enzymatic digestion and structure analysis of the fragment peptides were performed (Fig. 4*A*). The core N-terminal part [1–41] of synthetic BPP2 with three disulfide bonds was highly stable toward trypsin and Glu-C digestion, as with natural BPPs (Fig. S5). Thus, to conduct stepwise *S*-alkylation with limited reduction of disulfide bonds (38, 39), synthetic BPP2 denatured with 6 M aq. guanidine hydrochloride (Gdm-Cl)/0.1 M aq. citrate was treated with TCEP at 65 °C for 15 min, and *S*-alkylated with *N*-ethylmaleimide (NEM) at 27 °C for 20 min. The resulting peptide mixture was separated by RP-HPLC to afford four kinds of *S*-alkylated products (2SS-a/b and 1SS-a/b) as major products, in which one or two disulfide bonds were cleaved, respectively, along with unreacted BPP2 (3SS) and fully *S*-alkylated product (0SS) (Fig. 4*B*).

2SS-a and -b Having two NEM-Cys residues were then fully reduced with DTT, alkylated with IAM, and digested with Glu-C. MALDI-TOF MS/MS analysis of the digested peptides revealed that Cys^2^/Cys^27^ and Cys^9^/Cys^40^ were the original pair residues to form disulfide bonds in 2SS-a and -b, respectively (Figs. 4*C*, S6 and S7). Similarly, in 1SS-a and -b having four NEM–Cys residues, Cys^2^/Cys^27^ and Cys^6^/Cys^23^ were specifically carbamidomethylated, respectively. Furthermore, Glu-C digestion of 1SS-a and -b without further reductive *S*-alkylation gave the products with a disulfide bond at Cys^2^–Cys^27^ and Cys^6^–Cys^23^, respectively. Combining these results, the disulfide bond connectivity of synthetic BPP2 was established to be Cys(I)–Cys(V), Cys(II)–Cys(IV), and Cys(III)–Cys(VI), which was identical to the Type II. While the disulfide bond connectivity of natural BPPs 1 and 2 has not been determined directly, they were presumed to be the same as that of synthetic BPP2, based on the identity of the HPLC retention time and the high stability toward digestive enzymes, as mentioned above.

### Mealworm paralytic activity of BPP2

For the bioassay, injection of synthetic BPP2 (5.6 μg/g body weight) into the abdomen of mealworms (larva of *Z. atratus*) caused immediate lower-body paralysis (n = 3) (Table S1). This paralysis spread over the whole body within about 5 min and lasted for more than 1.5 h, with little responses to external stimuli. After 6 h, the mealworms gradually resumed locomotion and no lethality was observed for at least 2 days, although they remained sluggish in response to external stimuli. These characteristic symptoms on mealworms were similarly observed in all three specimens. Meanwhile, the lower doses (0.56 and 0.056 μg/g body weight) or PBS (used as a control) caused no significant effects (n = 3, each).

Shrews are known to paralyze and store prey such as earthworms and insects in their burrows (3, 18, 23, 24, 25), and in some cases, invertebrates paralyzed by bites have been shown to remain alive for several days (4). This observed phenomenon of keeping prey alive appears to be important for efficient capture and maintenance of their nutritional value. Thus, the paralytic effect of BPP2 would highly contribute to survival strategies of *Blarina* shrews in terms of predatory behavior and food storage.

### Electrophysiological analysis of BPP2

**Figure 5:**
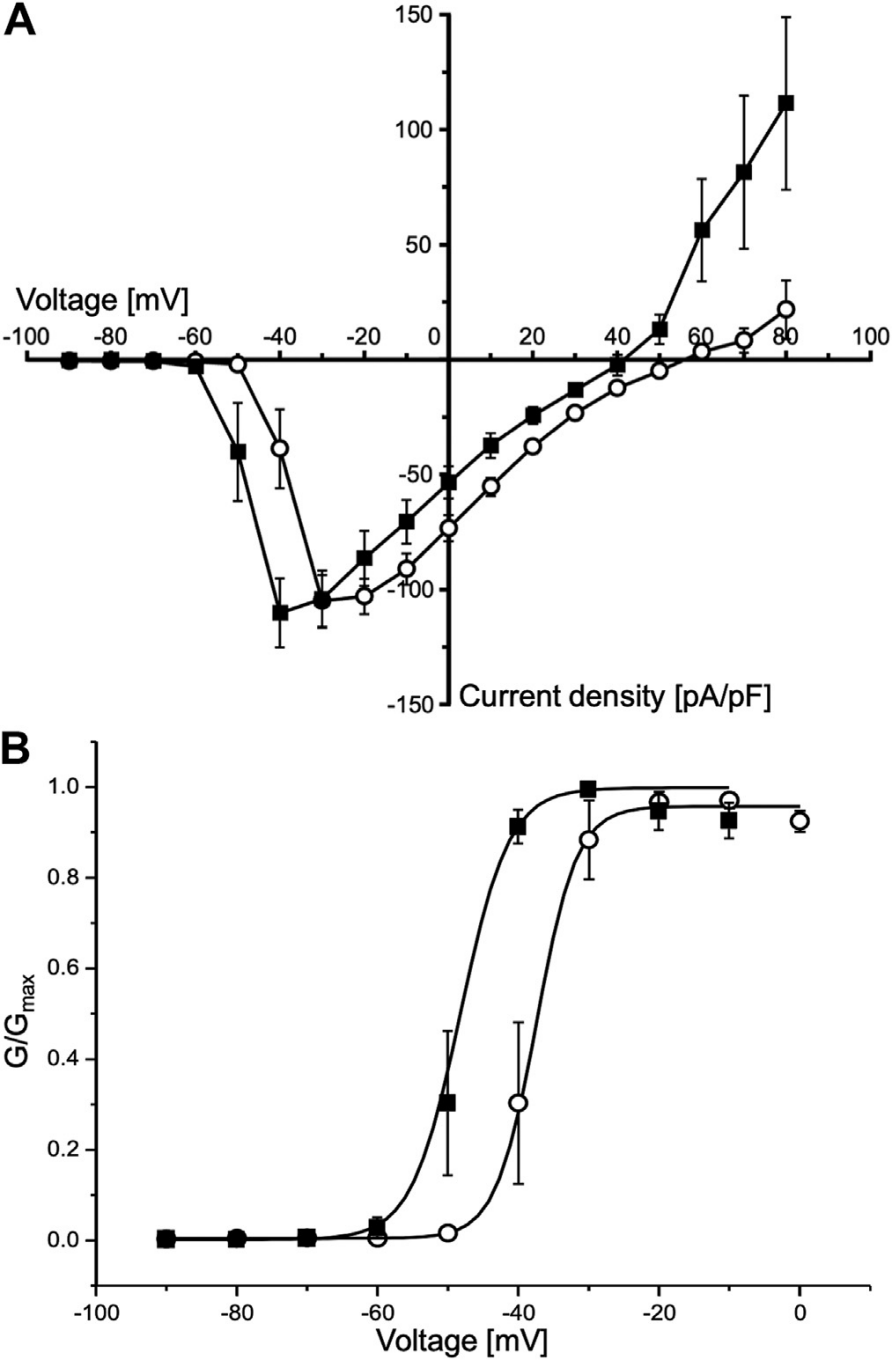
**Voltage-gated Ca**^**2+**^**currents measured by whole-cell patch-clamp experiments using HEK293 T cells expressing hCa**_**v**_**3.2.***A*, the current density–voltage relationships were recorded before (○) or after (▪) addition of synthetic BPP2 (0.84 μM, n = 5). *B*, steady-state activation curve of hCa_v_3.2. Normalized conductance (G/Gmax) was generated for both data sets, fit to a Boltzmann equation, and is shown as data ± S.E.M. (n = 5).

*Blarina* shrew venom causes severe pain to the bitten human (5). We expected that this venom targets Ca channels related to pain and neurotransmission, and thus first examined N-type Ca channel Ca_v_2.2, an important target for analgesia. In fact, several Ca_v_2.2 selective inhibitors, such as ω-conotoxin and its derivatives, have been investigated as pain-relief drugs (40), which have similar molecular sizes and three disulfide bonds with BPPs. However, no significant effects were observed on hCa_v_2.2 by the treatment with BPP2 up to 0.84 μM (Fig. S8). Thus, we next investigated a human T-type Ca^2+^ channel (hCa_v_3.2), which also initiates signal at the peripheral nerve endings in nociceptive pathways as with Ca_v_2.2 (41). Notably, synthetic BPP2 significantly activated hCa_v_3.2 at 0.84 μM (Fig. 5*A*). In whole-cell patch-clamp experiments, the peak of the lowest current density (*ca.* −110 pA/pF) was observed at −40 mV, being lower than those in control (*ca.* −105 pA/pF at −30 mV). In the steady-state activation curve, the midpoint of the activation curve was shifted by −11 mV compared to control (Fig. 5*B*). These results suggested that BPP2 increases sensitivity against the membrane potential change, similar to various gating modifier toxins (42, 43).

### Structural homology search of BPPs

In the CD spectrum of synthetic BPP2, two negative bands at 208 and 222 nm and a positive band at 195 nm were observed, which are characteristic for the α-helix structure (Fig. 3*D*) (44). This measured CD spectrum of synthetic BPP2 showed high agreement with the calculated (ColabFold-predicted) one by PDBMD2CD server (45), and their helical contents estimated by K2D3 server (46) were 55.7% and 54.9%, respectively (Fig. S9). To further investigate the structure–functionality relationship of BPPs, a homology search was performed using the Dali server (47) based on the stereostructure of BPP2 predicted by ColabFold (Fig. 2*C*). As a result, three structurally similar centipede neurotoxic peptides from *Scolopendra* sp., μ-SLPTX-Ssm6a (Na_v_1.7 inhibitor, PDB ID: 2mun), κ-SLPTX-Ssm1a (unspecified K_v_ inhibitor, PDB ID: 2m35), and κ-SLPTX-Ssd1a (KCNE1 inhibitor, PDB ID: 2mvt) (48, 49, 50), were nominated as the best ones in terms of structural similarity (RMSD = 1.6, 2.0 and 2.4 Å for 35∼37 C_α_ pairs, respectively) (Fig. S10). Despite a low sequence identity (∼19%), the disulfide bond connectivity of these SLPTXs was the same as that of BPP2 (Type II), which highly contributes to the fixation and stabilization of three helix structures.

## Discussion

On this work, we isolated BPPs 1 and 2 from the submaxillary glands of *B. brevicauda* and determined their structures. BPPs 1 and 2 have 47 and 52 amino acid residues and three disulfide bonds, which were established based on an analysis that combined partial reduction of the disulfide bonds of synthetic BPP2 and subsequent enzymatic digestion. As shown in Figure 1*C*, the submaxillary gland contained at least three BPPs, including an undetermined one with approximately 5.4 kDa. These concentrations were compared with that of synthetic BPP2 by the HPLC peak intensity. As a result, one individual of *Blarina* submaxillary gland contained approximately 100 μg of BPP2 and a total of 300∼400 μg of BPPs. Since 1 in 70 individuals with the venom extracts showed paralytic activity toward mealworms with 0.7 ∼ 1.0 g bodyweight, we estimated the effective dose of natural BPPs was 4.3 to 8.1 μg/g bodyweight, which was close to that of synthetic BPP2 (5.6 μg/g). While it cannot be accurately calculated by the BPP2 activity alone, the characteristic symptom of paralyzed mealworms with synthetic BPP2 was highly similar to that of those with the crude venom extract. Thus, we concluded that BPP2 was important and responsible for a paralytic substance of *Blarina* shrew.

Synthetic BPP2 showed mealworm paralytic activity and negatively shifted the activation a human T-type Ca^2+^ channel (hCa_v_3.2). hCa_v_3.2 Also plays an important role in repetitive firing in the brain and heart, and several inhibitors have been identified, such as a carbazole derivative NMP-7 (51, 52) and the absence seizure drug ethosuximide (53). Meanwhile, as for T-type Ca^2+^ channel activators, the spiroimidazopyridine derivative SAK3 specifically enhances hCa_v_3.1 and hCa_v_3.3, which is expected to be a drug lead for Alzheimer’s disease, but not hCa_v_3.2 (54, 55). Therefore, to the best of our knowledge, BPP2 is the first molecule to potentially activate hCa_v_3.2. BPPs could also cause voltage-gated Ca2+ channels (VGCC)-related pain, peripheral nerve hyperesthesia, and other dysfunctions in the mammalian nervous system. This discovery should help us to develop new specific VGCC modulators and to understand new mechanisms of action in diseases such as hypersensitivity and neurodegenerative diseases.

There is no direct evidence that T-type Ca channel activation of BPP2 is associated with paralytic activity of mealworms. However, it was shown that T-type Ca channels are involved in muscle cell contraction of nematode (*Caenorhabditis elegans*) (56) and that mutation of CCA-1, a T-type Ca channel homolog expressed in the pharyngeal muscle, inhibits the pharyngeal pump (feeding movement) of *C. elegans* (57). These results suggested that T-type Ca channels play an important role in invertebrate locomotion. Since T-type Ca^2+^ channels in invertebrates and mammals have similar structural and biophysical properties (58, 59), the specific Ca^2+^ channel activation property of BPP2 might also be related to its mealworm paralytic activity.

SLPTXs have structural features and disulfide bond connectivity similar to those in ion transport peptides (ITP), non-toxic hormones that belong to the crustacean hyperglycemic hormone (CHH)/ITP superfamily, and are widely conserved in arthropods, insects, and crustaceans. It has also been reported that SLPTXs used for predation were derived from a common ancestor of CHH/ITP peptides based on a transcriptome analysis of the venom glands (60). In addition, the C-terminal region of CHH/ITP peptides is important for their hormonal activity (61), whereas SLPTXs lack this moiety. These structural differences have been suggested to be important for the functions of these centipede toxins. BPPs have high sequence similarity to synenkephalins, which are widely conserved endogenous peptides in several mammalian organs, including brain and adrenal gland (62, 63, 64, 65), while there have been no reports on their presence in saliva or salivary glands, except for several shrews and solenodons (11, 12, 19). Thus, as with SLPTXs, convergent evolution of endogenous peptides might provide BPPs, which help *Blarina* shrews to capture prey efficiently. Our study also revealed that the disulfide bond connectivity of BPPs (Type II) was different from those of reported rat recombinant synenkephalins (Type I), as were lengths of their C-terminal residues. These results suggested that hSYN and other mammalian synenkephalins in the brain might have different functions than BPPs in the saliva. From ecological and evolutionary viewpoints, shrews have weaponized endogenous (perhaps non-toxic) peptides and used them in saliva as paralytic venoms for predation. In other words, it is interesting that mammalian synenkephalins may exert unique functions if the extra C-terminal sequence corresponding to the pre-pro regions of enkephalin is cleaved and further secreted into saliva, as for BPPs. There are many examples of widely conserved endogenous proteins and peptides that have acquired toxicity during the evolution of venomous animals (6, 7), such as snakes (66, 67, 68), scorpions (69), and cone snails (70). Our finding that the homologs of endogenous peptides widely conserved in mammals were secreted as neurotoxins in shrew saliva is an excellent example of the chemical evolution of venom and the evolution of mammals as a species.

BPPs have a stereostructure and disulfide bond connectivity similar to those of SLPTXs, which exhibit lethal activity against insects and ion channel inhibitory activity. It has been suggested that these centipede peptides evolved from a common ancestor with endogenous peptide hormones that are widely conserved in arthropods. As with the centipede venoms, BPPs might result from convergent evolution in which some endogenous peptides are weaponized for efficient predation. From both ecological and evolutionarily perspectives, it is highly interesting to investigate the structure-activity relationship among BPPs and other mammalian synenkephalins, as well as the differences in their mechanisms of action. Thus, our study provides new insights for understanding the great mystery behind the structure and function of mammalian venoms, namely, the survival strategies and evolution of venomous animals.

## Experimental procedures

### Tissue sample storage and extraction of BPPs

The Northern short-tailed shrews *Blairna brevicauda* were captured in July 2002 and September 2003 within the Fresh Air Camp and the E. S. George Reserve, University of Michigan (Livingstone Country, MI, USA), and tissue samples were stored in acetone at −20 °C, as described previously (20). Submaxillary glands (approximately 30 mg per individual after acetone removal) were homogenized in 0.85% NaCl aq, and the suspensions were centrifuged (15,000 rpm, 4 °C, 30 min) to give the crude extracts.

### Purification of natural BPPs 1 and 2

#### Wide pore RP-HPLC

Wide pore RP-HPLC was performed with a Develosil 300C4-HG-5 (5 μm, ϕ 4.6 mm × 250 mm) at 4 °C with a linear gradient of 10 to 60% aq. MeCN/0.05% TFA for 60 min, flow rate at 0.5 ml/min (Figs. 1*A* and S1, *A* and *B*). Monitoring at UV 215 nm.

#### Gel-permeation HPLC

Gel-permeation HPLC was performed with a TSKgel G2000SWXL (5 μm, ϕ 7.8 mm × 300 mm, Tosoh, Tokyo, Japan) at 20 °C with a linear gradient of 20% to 80% aq. MeCN/0.05% TFA for 80 min, flow rate at 0.2 ml/min. Monitoring at UV 280 nm (Fig. 1*B*).

#### C_30_ RP-HPLC

C_30_ RP-HPLC was performed with a Develosil RP-AQUEOUS AR-5 (5 μm, ϕ 4.6 mm × 250 mm) at 23 °C with a linear gradient of 20% to 50% aq. MeCN (containing with 0.1–0.05% HFBA) for 60 min, flow rate at 0.5 ml/min (Figs. 1*C* and S1*C*). Monitoring at UV 280 nm. MS (MALDI-TOF): natural BPP1 *m*/*z* 5092.8 (calculated for C_215_H_339_N_58_O_73_S_6_^+^ [M + H]^+^, Δ +0.5 mu), natural BPP2 *m*/*z* 5646.0 (calculated for C_241_H_383_N_64_O_80_S_6_^+^ [M + H]^+^, Δ +0.4 mu).

### Amino acid sequence analysis of BPPs 1 and 2

#### Trypsin digestion

To a solution of natural BPPs 1 and 2 in 50 mM aq. NH_4_HCO_3_ (25 μl) were dissolved with 45 mM DTT in 25 mM NH_4_HCO_3_ (0.5 μl) (Figs. 2*A*, S2 and S3). After incubation at 50 °C for 15 min, 100 mM IAM in 25 mM aq. NH_4_HCO_3_ (0.5 μl) was added at room temperature. After 15 min, trypsin (50 ng in 0.5 μl of 50 mM acetic acid, Promega) was added, and the resulting mixture was incubated at 37 °C overnight. The reactions were quenched with 10% aq. TFA (0.6 μl).

#### Glu-C digestion

Reduction and alkylation of BPPs 1 and 2 were performed as mentioned above. Glu-C (50 ng in 0.5 μl of DDW, Promega) was added to the solution, and the resulting mixture was incubated at 37 °C overnight. The reactions were quenched with 10% aq. TFA (0.6 μl).

The digested peptides were desalted with a Ziptip C18 tip column (Millipore, Billerica, USA) and eluted with α-CHCA as a matrix and analyzed by MALDI-TOF MS and MS/MS.

### Disulfide bond connectivity analysis of BPP2

Disulfide connectivity analysis of synthetic BPP2 was performed with a partial reduction and alkylation method (38, 39) (Figs. 4, S6, and S7).

#### Partial reduction and NEM-alkylation

Synthetic BPP2 (25 μg, 4.4 nmol) was denatured with 6 M Gdm-Cl in 0.1 M citrate (4.4 μl, pH 3.0) at 65 °C for 20 min. Subsequently, 10 mM TCEP·HCl in the same buffer (1.3 μl, pH: 3.0) was added. After incubating at 65 °C for 15 min, 0.2 M NEM in 0.1 M citrate (5.3 μl) was added. The resulting mixture was incubated at 27 °C for 20 min and directly purified by RP-HPLC using a Develosil ODS-HG-5 column (5 μm, ϕ 4.6 mm × 250 mm) at 25 °C with an isocratic 20% aq. MeCN/0.1% TFA for 5 min then a linear gradient 20% to 50% aq. MeCN/0.1% TFA for 60 min, at a flow rate of 1.0 ml/min to give 3SS, 2SS-a/b, 1SS-a/b, and 0SS, which were concentrated and lyophilized for further degradation reactions.

#### Full reduction, Cam-alkylation, and enzymatic digestion

The NEM-alkylated peptides were treated with 45 mM DTT in 52.6 mM aq. NH_4_HCO_3_ (2.0 μl, pH: 7.0). After incubation at 50 °C for 15 min, 0.1 M IAM in 52.6 mM aq. NH_4_HCO_3_ (1.0 μl) was added. After incubation at 27 °C for 15 min, the resulting mixture was diluted with 52.6 mM aq. NH_4_HCO_3_ (16 μl) and treated with Glu-C (50 ng in 1.0 μl of DDW) at pH 7.0, 37 °C, for 3 h. The resulting peptide mixture was directly analyzed with MALDI-TOF MS and MS/MS without a desalting process.

### Circular dichroism spectrometry

Circular dichroism (CD) spectrum was measured by a JASCO J-1500 CD spectrometer at 25 °C. Synthetic BPP2 was dissolved in 50 mM KHPO_4_, pH 7.0 (final concentration: 20 μM) in a 1 mm pathlength cell (Fig. 3*D*). The spectrum consisted of five scans acquired with a scan rate of 20 nm/min and a digital integration time of 2 s. A spectrum of the buffer recorded under the same conditions was subtracted from that of the sample. The percent helicity was calculated from the molar CD using the K2D3 web server (45).

### Mealworm bioassay

Paralytic activity against mealworms was examined by using synthetic BPP2 dissolved in saline with mealworms (larva of *Z. atratus*, body weight: 0.7 ∼ 1.0 g). Synthetic BPP2 (5.6, 0.56, and 0.056 μg/g body weight) was injected into the abdomen of mealworms and observed immediate lower-body paralysis (Table S1). In brief, 10, 1, and 0.1 μM solutions of BPP2 in PBS were used for paralytic assay (injection volume: 100 μl per 1.0 g mealworm bodyweight). Phosphate buffered saline (PBS) was used as a control.

### Electrophysiology

Electrophysiological assays were done on human Ca_v_3.2 or Ca_v_2.2 channels, which were expressed on Human embryonic kidney 293T (HEK293T) cells. Voltage-gated Ca^2+^ currents were recorded by the whole-cell patch-clamp technique, as previously described (71). This recording was performed in the whole-cell configuration using an Axopatch 200B Amplifier (Axon CNS, Molecular Devices) at a holding potential of −90 mV. Cells were depolarized from the −90 mV holding potential to +80 mV with +10 mV voltage steps of 200 ms. The current amplitude was measured at the peak. The current density was determined with the cell capacitance to establish the density-voltage curves. The currents were filtered at 2 kHz and sampled at 5 kHz using an A/D converter, the Digidata 1440A (Axon CNS, Molecular Devices). The leak was subtracted automatically by a P/4 protocol (pclamp10, Axon CNS, Molecular Devices). For the recording Ca^2+^ currents, the bath solution contained 125 mM *N*-methyl-d-glucamine, 5 mM 4-aminopyridine, 20 mM tetraethyl-ammonium chloride, 2 mM CaCl_2_, 2 mM MgCl_2_, and 10 mM d-glucose and was buffered to pH 7.4 with 10 mM HEPES. The patch pipettes were filled with solution containing 130 mM CsCl, 10 mM EGTA, 3 mM Mg-ATP, and 0.4 mM Li-GTP with the pH adjusted to 7.2 by 25 mM HEPES.

## Data availability

The research data supporting this publication are provided within this article, or as supplementary information.

## Supporting information

This article contains supporting information (28, 32, 33, 36, 37, 45, 46, 47, 72, 73, 74, 75, 76).

## Conflict of interest

The authors declare that they have no conflicts of interest with the contents of this article.
