# Supplementary material for: Mammalian neurotoxins, Blarina paralytic peptides, cause hyperpolarization of human T-type Ca channel hCav3.2 activation
Source: J Biol Chem. 2023 Jul 17;299(9):105066. doi: 10.1016/j.jbc.2023.105066 (PMC10493266; doi:10.1016/j.jbc.2023.105066)
Supplement: Supporting Information [file mmc1.pdf]

Supporting information

Mammalian neurotoxins, *Blarina* paralytic peptides, cause hyperpolarization of human T-type Ca channel hCa$_v$3.2 activation

Yusuke Yano,[1] Ryo Fukuoka,[1] Andres D. Maturana,[1] Satoshi D. Ohdachi,[2] and Masaki Kita[1,]*

1. Graduate School of Bioagricultural Sciences, Nagoya University, Furo-cho, Chikusa, Nagoya 464-8601, Japan.
2. Institute of Low Temperature Science, Hokkaido University, Kita-19 Nishi-8, Kita-ku, Sapporo 060-0819, Japan.

* Corresponding author: Prof. Masaki Kita (e-mail: mkita@agr.nagoya-u.ac.jp)

Running title: BPPs cause a hyperpolarization of human Ca$_v$3.2 activation

Keywords: Mammalian venom, Neurotoxin, Peptide chemical synthesis, Disulfide, Calcium channel, Molecular evolution, Gating modifier toxin

Table of Contents

Abbreviations S2

General information S3

Supporting procedures S4

1. BLAST search
2. Stereostructure prediction of BPP2
3. Peptide synthesis

Supporting figures S10
Supporting table S34

Abbreviations

acac	acetylacetone
α-CHCA	α-cyano-4-hydroxycinnamic acid
Boc	*tert*-butoxycarbonyl
Cam	carbamidomethyl
CHH	crustacean hyperglycemic hormone
DIC	*N,N'*-diisopropylcarbodiimide
DMF	*N,N*-dimethylformamide
Fmoc	9-fluorenylmethyloxycarbonyl
Gdm-Cl	guanidine hydrochloride
Glu-C	glutamyl endopeptidase
HCTU	5-Chloro-1-((dimethylamino)(dimethyliminio)methyl)-1*H*-benzo[*d*][1,2,3]triazole 3-oxide hexafluorophosphate
HFBA	heptafluorobutyric acid
HOBt	hydroxybenzotriazole
IAM	iodoacetamide
ITP	ion transport peptides
LDDT	local distance difference test
MALDI-TOF	matrix-assisted laser desorption / ionization time-of-flight
MPAA	4-mercaptophenylacetic acid
NCL	native chemical ligation
NEM	*N*-ethylmaleimide
NMM	*N*-methylmorpholine
Pbf	2,2,4,6,7-Pentamethyldihydrobenzofuran-5-sulfonyl
SPPS	solid-phase peptide synthesis
*t*Bu	*tert*-butyl
TCEP·HCl	tris(2-carboxyethyl)phosphine hydrochloride
TIPS	triisopropylsilane
Trt	trityl
VGCCs	voltage-gated Ca^{2+} channels

General information

Chemicals

All reagents and solvents were purchased from Merck KGaA (Darmstadt, Germany), Sigma-Aldrich (Sigma Chemical Co., St Louis, MO), AnaSpec Inc. (Fremont, CA, USA), Kanto Chemical Co., Inc. (Tokyo, Japan), Tokyo Chemical Industry Co., Ltd. (Tokyo, Japan), FUJIFILM Wako Pure Chemical Corporation (Osaka, Japan), and Nacalai Tesque (Kyoto, Japan). All chemicals were used as obtained commercially unless otherwise noted.

Mass spectrometry

MALDI-TOF MS and MS/MS analysis were performed using a Bruker UltrafleXtreme spectrometer. A saturated solution of α-CHCA in 50% aq. MeCN / 0.1% TFA was used as a matrix.

HPLC

Analysis and purification of all peptides were performed with a JASCO semi-micro HPLC system fitted 200 μL sample loop consisting of dual pumps (PU-2085 Plus), a mixer (MX-2080), a degasser (DG-2080-53), a UV detector (MD-2018), a column thermostat (CO-2060 Plus) or on a JASCO preparative HPLC system fitted with a 1 mL sample loop consisting of a pump (PU-2089 Plus), a UV detector (UV-4075), a fraction collector (ADVANTEC CHF122SC) and a recorder (807-IT). Develosil® series columns (Nomura Chemical Co., Ltd., Aichi, Japan) were used for RP-HPLC, and a TSKgel® G2000SW$_{XL}$ column (Tosoh, Tokyo, Japan) was used for gel-permeation HPLC.

Supporting procedures

1 BLAST search

A similarity search for the amino acid sequence of BPPs was performed with the protein BLAST (72) on the UniProt website. The amino acid sequence of BPP2 was used as the query sequence, and the UniProtKB reference proteomes + Swiss-Prot was used for the sequence search. The Clustal omega program (73) was performed for the peptide sequence alignment.

2 Stereostructure prediction of BPP2

Stereostructure prediction of BPP2 was performed by ColabFold (74) (a Google Colab version of AlphaFold2 (75) using MMSeq2 (76)), without any template structures. Generated five predicted structures were evaluated by the pLDDT score (77). All peptide structures were visualized by Chimera X (78) in Fig. S4. The highest structure in the pLDDT value is shown in Fig. 2C.

3 Peptide synthesis

3.1 Automated Solid Phase Peptide Synthesis (SPPS)

Peptides were synthesized on a CSBio 136X synthesizer using a standard Fmoc SPPS chemistry. The following Fmoc amino acids with the protected side-chains were used: Fmoc-Ala-OH, Fmoc-Arg(Pbf)-OH, Fmoc-Asn(Trt)-OH, Fmoc-Asp(O*t*Bu)-OH, Fmoc-Cys(Trt)-OH, Fmoc-Gln(Trt)-OH, Fmoc-Glu(O*t*Bu)-OH, Fmoc-Gly-OH, Fmoc-His(1-Trt)-OH, Fmoc-Ile-OH, Fmoc-Leu-OH, Fmoc-Lys(Boc)-OH, Fmoc-Phe-OH, Fmoc-Pro-OH, Fmoc-Ser(*t*Bu)-OH, Fmoc-Thr(*t*Bu)-OH, Fmoc-Trp(Boc)-OH, Fmoc-Tyr(*t*Bu)-OH, and Fmoc-Val-OH. SPPS was performed with a 2-chlorotrityl chloride resin or an H-Thr(*t*Bu)-hydrazide preloaded 2-chlorotrityl resin. The first residue was manually loaded onto the resin and subsequent Fmoc-SPPS followed the standard protocols. Fmoc-deprotections were performed with 20% (v/v) piperidine in DMF (8 min × 2). Couplings were performed with Fmoc-amino acid (4.0 equiv relative to resin substitution), HCTU (3.8 equiv) and NMM (8.0 equiv) in DMF for 60 min. If required, the coupling step was repeated (double coupling) and LiCl washes (0.8 M LiCl in DMF) were performed before the next step. After coupling, unreacted free amine was capped by treatment with 20% (v/v) acetic anhydride and 10% (v/v) NMM in DMF for 10 min. For the coupling with the residues prone to epimerization such as cysteine, HOBt (4.0 equiv) and DIC (4.0 equiv) were in DMF were used, and the reaction time was extended to 2 h. After the peptide elongation, the resin was washed several times with DMF followed by CH_2Cl_2, dried and treated with a TFA cleavage cocktail (TFA/phenol/H_2O/TIPS = 88/5/5/2, vol/wt/vol/vol, 30 mL/g resin) for 3 h at room temperature. After filtration, the filtrate was concentrated under reduced pressure. The residue was triturated with cooled Et_2O, centrifuged and the supernatant was removed by decantation. This trituration/decantation step was repeated three times. The obtained crude peptide was dissolved in 0.1 % aq. TFA, lyophilized, and purified with RP-HPLC.

3.2 Synthesis of N-terminal segment [1-22]

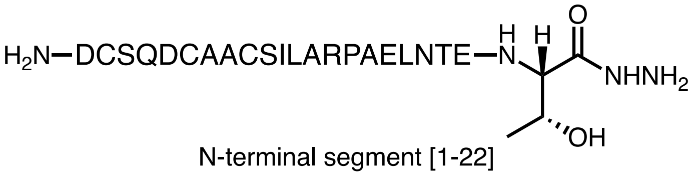

The N-terminal segment [1-22] was synthesized on the H-Thr(*t*Bu)-$NHNH_2$ preloaded 2-chloro trityl resin (500 mg, 0.18 mmol, loading: 0.35 mmol/g) by automated Fmoc SPPS. The crude peptide (1/8 amount) was purified by RP-HPLC using a Develosil® ODS HG-5 column (5 μm, ϕ 20 mm x 250 mm) at room temperature with an isocratic 25% aq. MeCN/0.1% TFA for 10 min and a linear gradient of 25–50% aq. MeCN / 0.1% TFA for 75.6 min, at a flow rate of 7.5 mL/min to N-terminal segment [1-22] (9.1 mg, 17% in relative yield).
t_R: 9.4 min [Develosil® ODS HG-5 column (5 μm, ϕ 4.6 mm x 250 mm) at 25 ºC with a gradient of 25–55% aq. MeCN / 0.1% TFA in 30 min, flow rate 1.0 mL/min, see below]. MS (MALDI-TOF): *m/z* 2325.1 (calculated for $C_{91}H_{154}N_{29}O_{36}S_3^+$ $[M+H]^+$, Δ +0.1 mu).

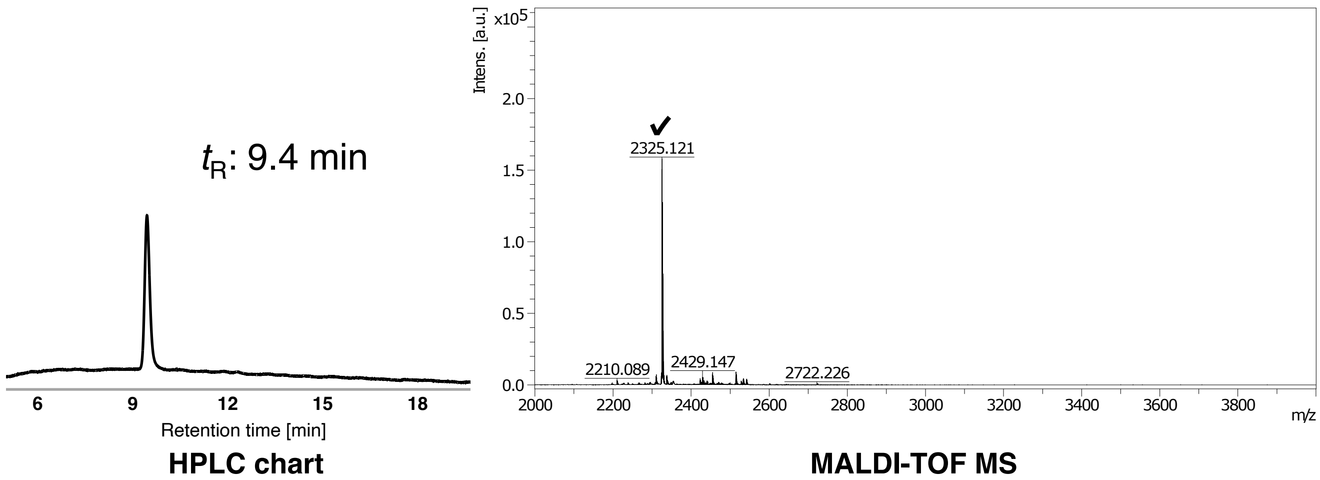

3.3 Synthesis of C-terminal segment [23-52]

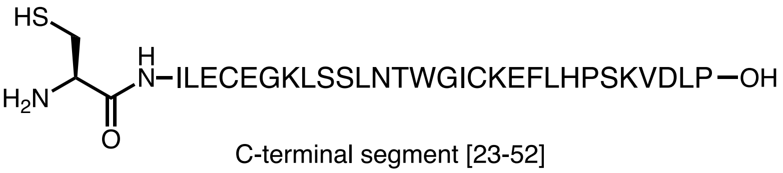

C-terminal segment [23-52]

The C-terminal segment [23-52] was synthesized on the Fmoc-Pro-OH preloaded 2-chloro trityl resin (1.0 g, 0.29 mmol, loading: 0.29 mmol/g) by automated Fmoc SPPS. The crude peptide (1/5 amount) was purified by RP-HPLC using a Develosil® ODS HG-5 column (5 μm, ϕ 20 mm x 250 mm) at room temperature with an isocratic 30% aq. MeCN / 0.1% TFA for 10 min then a linear gradient of 30–50% aq. MeCN / 0.1% TFA in 75.6 min, at a flow rate of 7.5 mL/min to C-terminal segment [23-52]. (19.5 mg, 10% in relative yield).

t_R: 18.9 min [Develosil® ODS HG-5 (5 μm, ϕ 4.6 mm x 250 mm) at 25 ºC with a linear gradient of 25–55% aq. MeCN / 0.1% TFA for 30 min, flow rate 1.0 mL/min, see below]. MS (MALDI-TOF): *m/z* 3359.7 (calculated for $C_{150}H_{240}N_{37}O_{44}S_3^+$ $[M+H]^+$, Δ ±0.0 mu)

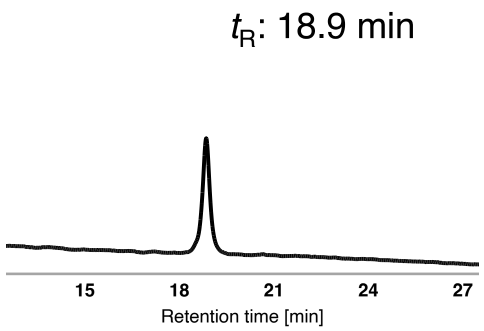

HPLC chart

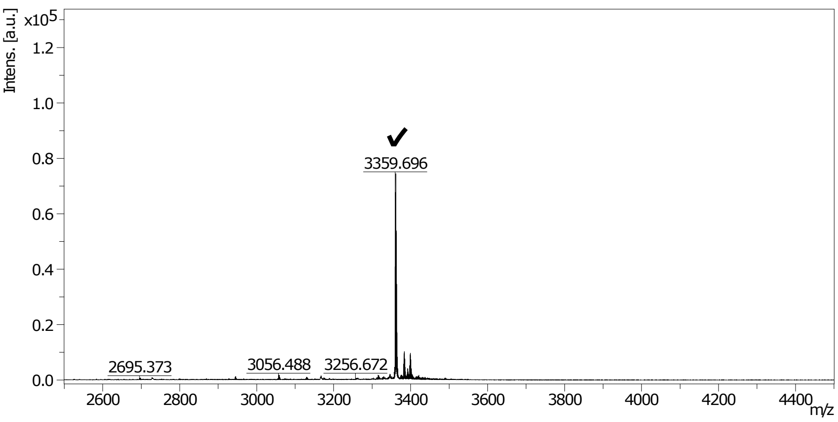

MALDI-TOF MS

3.4 Synthesis of linear BPP2

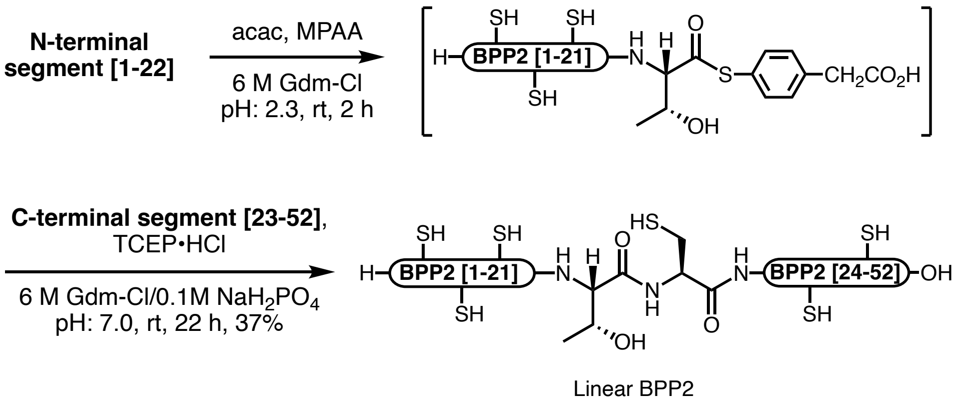

The prepared two segments were condensed by using a native chemical ligation via an acylpyrazole intermediate (79). N-terminal segment [1-22] (5.8 mg, 2.4 μmol) was dissolved in 6 M Gdm-Cl (270 μL, pH 3.0), then 1 M acetylacetone / 6 M aq. Gdm-Cl (23.5 μL, 23.5 μmol) and MPAA (9.9 mg, 59 μmol) were added. After stirring at room temperature for 2 h, C-terminal segment [23-52] (9.0 mg, 2.6 μmol), and TCEP·HCl (4.2 mg, 15 μmol) dissolved in 6 M Gdm-Cl / 0.2 M NaH_2PO_4 (294 μL, pH 8.5) were added, and adjusted to pH 7.0 with 6 M aq. NaOH (5.0 μL). After standing at room temperature for 23 h, the reaction mixture was quench with 20 mM TCEP·HCl (1 mL), and purified by RP-HPLC using a Develosil® ODS HG-5 column (5 μm, ϕ 20 mm x 250 mm) at room temperature with an isocratic 20% aq. MeCN / 0.1% TFA for 10 min then a linear gradient of 20–50% aq. MeCN / 0.1% TFA for 75.6 min, at a flow rate of 7.5 mL/min to give linear BPP2 (4.9 mg, 37%).

t_R: 21.5 min [Develosil® ODS HG-5 (5 μm, ϕ 4.6 mm x 250 mm) at 25 ºC with a linear gradient of 25–55% aq. MeCN / 0.1% TFA for 30 min, flow rate 1.0 mL/min, see below]. MS (MALDI-TOF): *m/z* 5652.1 (calculated for $C_{241}H_{389}N_{64}O_{80}S_6^+$ $[M+H]^+$, Δ +0.4 mu)

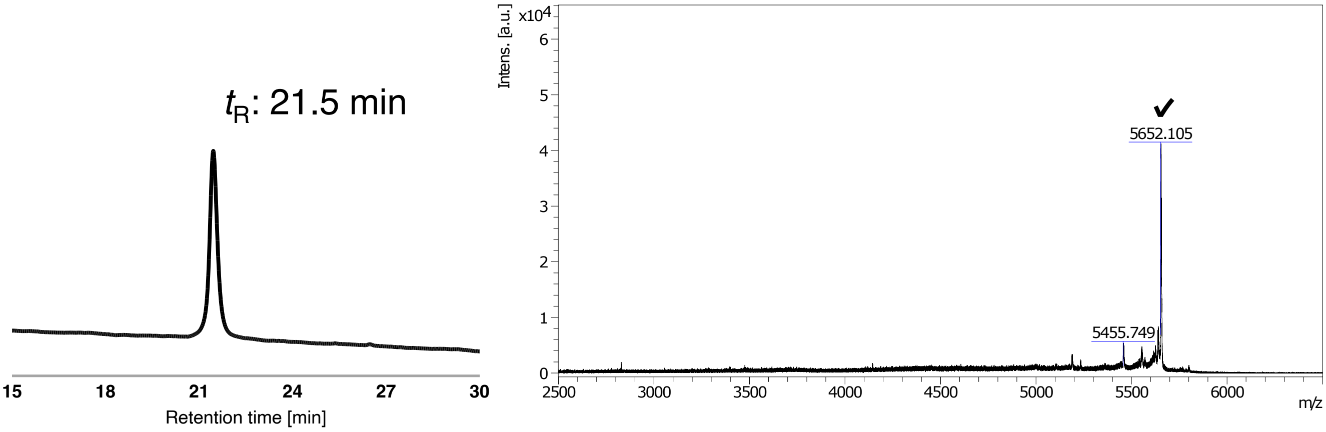

HPLC chart MALDI-TOF MS

3.5 Synthesis of BPP2

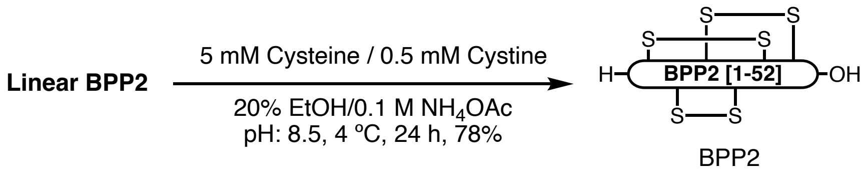

Refolding of the linear BPP2 was conducted using a cysteine/cystine redox system. The linear BPP2 (1.3 mg, 0.21 μmol) was dissolved in the refolding buffer (80) (5 mM cysteine / 0.5 mM cystine in 20% EtOH / 0.1 M aq. NH_4OAc, pH 8.5, 4.1 mL). After stirring at 4 ºC for 24 h, the reaction mixture was lyophilized, and purified by RP-HPLC using a Develosil® ODS HG-5 column (5 μm, ϕ 4.6 mm x 250 mm) at 25 ºC with a linear gradient of 25–50% aq. MeCN / 0.1% TFA for 25 min, at a flow rate of 1.0 mL/min to afford BPP2 (1.0 mg, 78%).

t_R: 13.8 min [Develosil® ODS HG-5 (5 μm, ϕ 4.6 mm x 250 mm) at 25 ºC with a linear gradient of 25–55% aq. MeCN / 0.1% TFA for 30 min, flow rate 1.0 mL/min, see below]. MS (MALDI-TOF): *m/z* 5644.9 (calculated for $C_{241}H_{383}N_{64}O_{80}S_6^+$ $[M+H]^+$, Δ –0.7 mu).

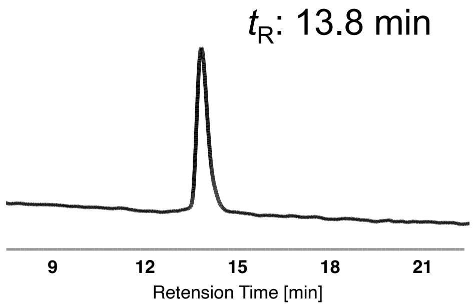

HPLC chart

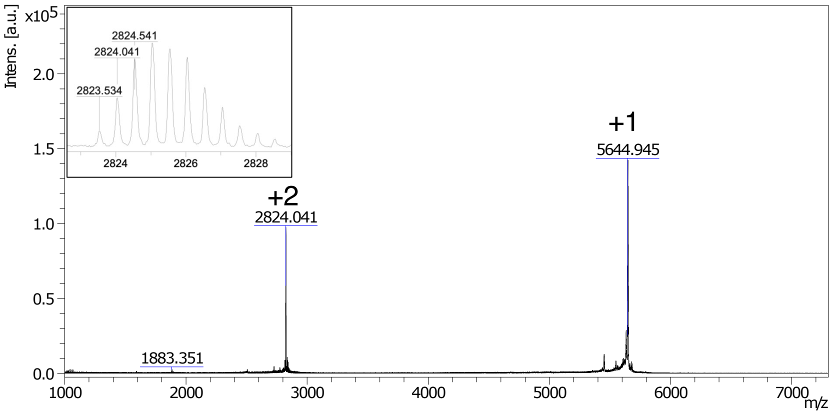

MALDI-TOF MS

Comparison of the retention times of natural and synthetic BPP2 was performed by RP-HPLC using a Develosil® RP-AQUEOUS AR-5 column (C30, 5 μm, ϕ 4.6 mm x 250 mm) at 23 ºC with a linear gradient of 20–50% aq. MeCN (containing 0.054%–0.060% HFBA) for 60 min, at a flow rate 0.5 mL/min (Fig. 3C).

Supporting figures

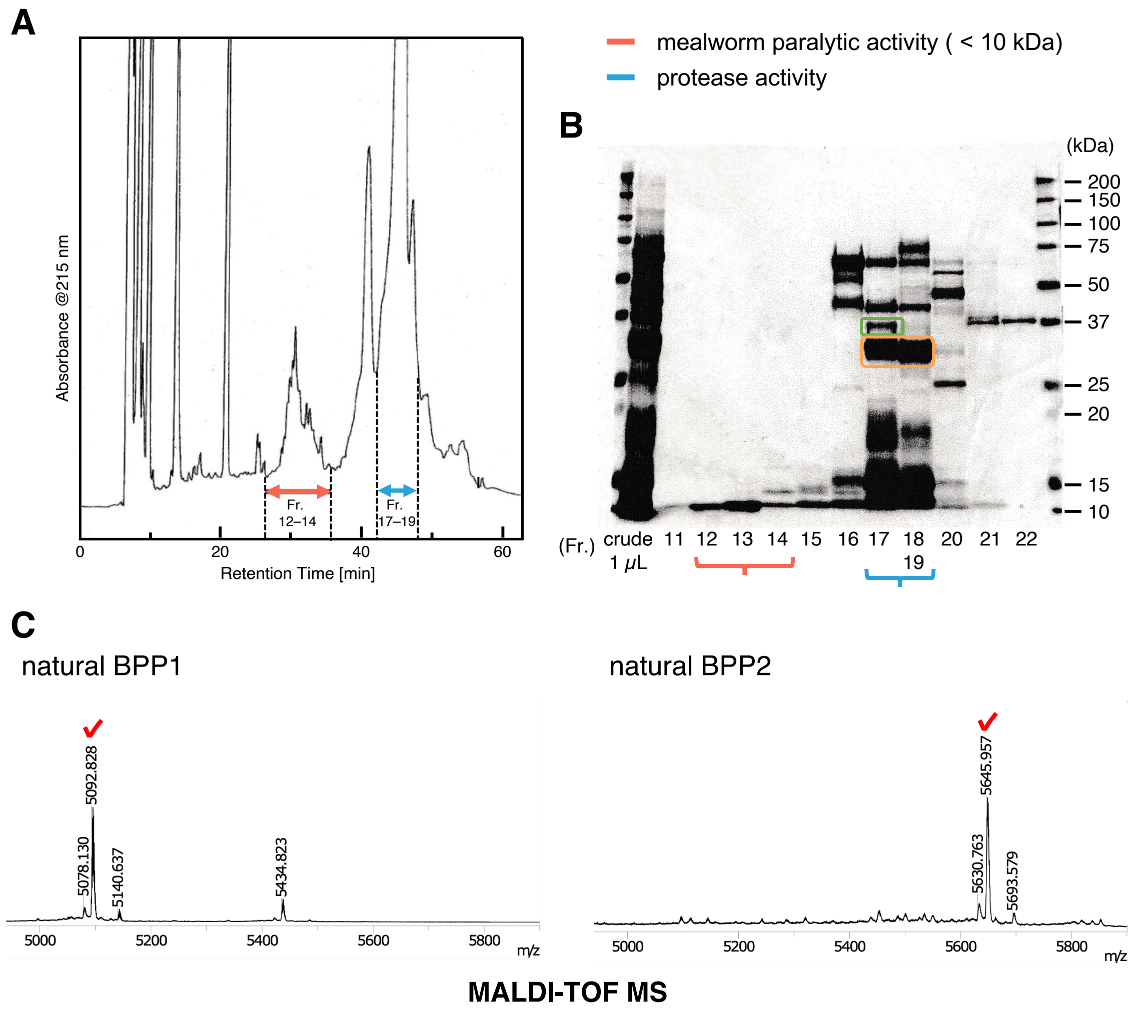

Figure S1. Separation of lower or higher-molecular-weight fractions of the extracts of *Blarina brevicauda* submaxillary glands. **A**: A chromatogram of wide-pore (300 Å) C_4 column RP-HPLC (same as Figure. 1A). Red bar shows the fractions with mealworm paralytic activity, and the blue bar shows the fractions with protease activity. **B**: SDS-PAGE analysis of separated fractions. Two kallikrein-like proteases, BLTX and blarinasin, are shown in orange and green squares, respectively. **C**: MALDI-TOF MS analysis of isolated natural BPP1 and BPP2.

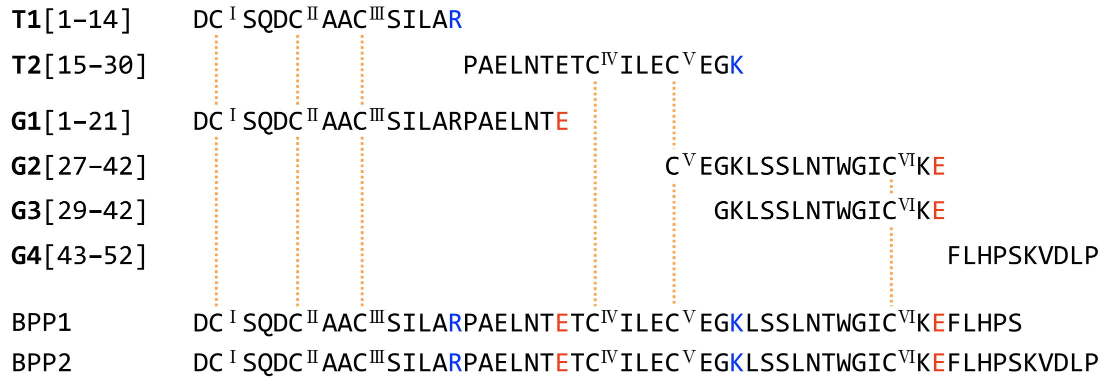

	position	$[M+H]^+_{Calc.}$	$[M+H]^+_{Exp.}$	Identified fragments
		m/z		
**T1**	[1–14]	1683.7	1683.7	DC^{I}(Cam)SQDC^{II}(Cam)AAC^{III}(Cam)SILAR
**T2**	[15–30]	1920.9	1920.8	PAELNTETC^{IV}(Cam)ILEC^{V}(Cam)EGK
**G1**	[1–21]	2381.0	2381.0	DC^{I}(Cam)SQDC^{II}(Cam)AAC^{III}(Cam)SILARPAELNTE
**G2**	[27–42]	1881.8	1881.9	C^{V}(Cam)EGKLSSLNTWGIC^{VI}(Cam)KE
**G3**	[29–42]	1592.8	1592.7	GKLSSLNTWGIC^{VI}(Cam)KE
**G4**	[43–52]	1152.6	1152.6	FLHPSKVDLP

Figure S2. Digested peptide fragments of natural BPPs 1 and 2. **T1–2**: trypsin digested peptide fragments. **G1–4**: Glu-C digested peptide fragments. Cysteine residues were *S*-carbamidomethylated. Monoisotopic masses are used for calculated and measured *m/z*. MALDI-MS/MS of the digested peptides from BPP2 are shown in Fig. S3. The same digested peptides (**T1**, **T2**, **G1–3**) of BPP1 were also established by MS/MS analysis.

Figure S3-1

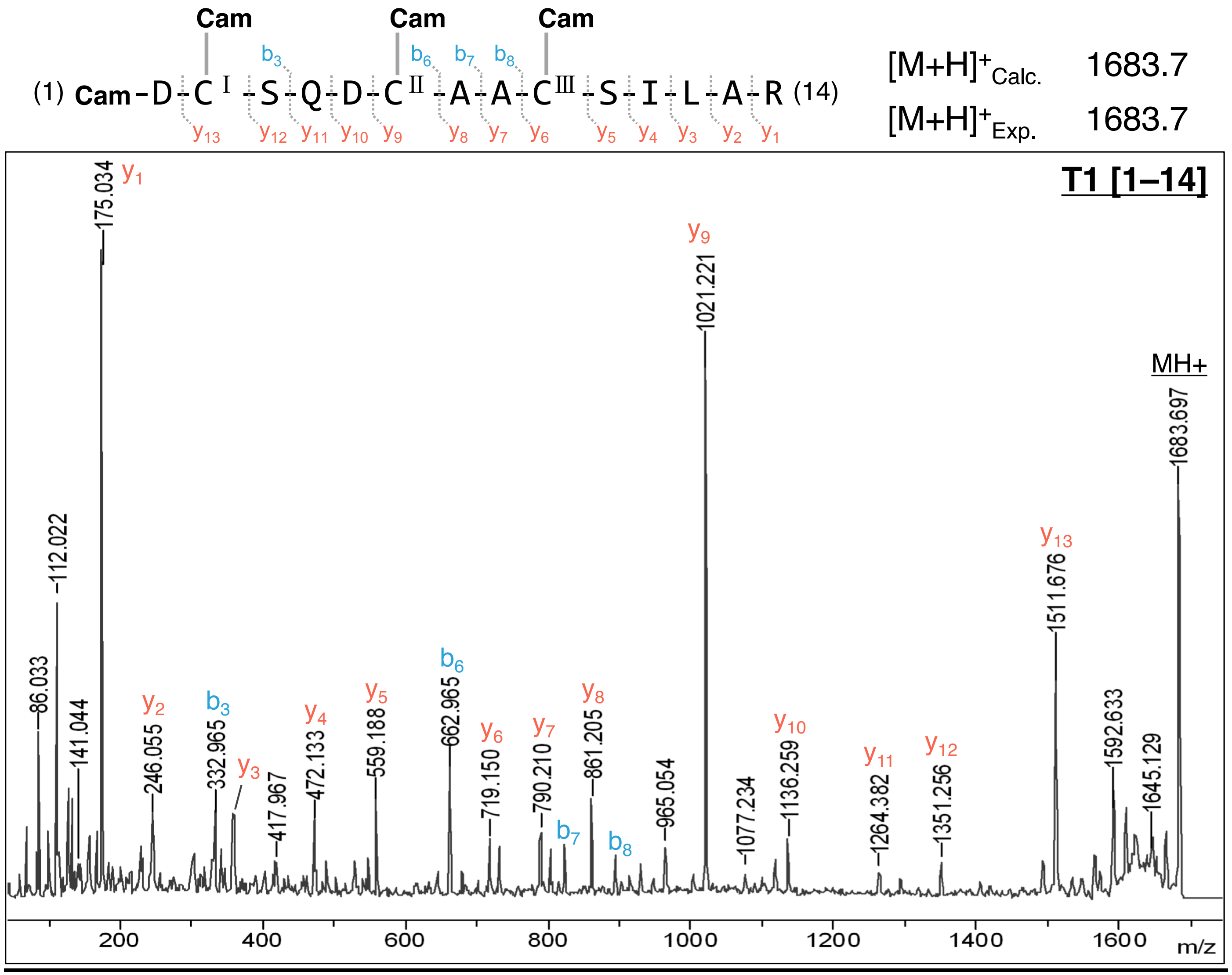

Cam
Cam
Cam
(1) Cam-D-C I -S-Q-D-C II -A-A-C III -S-I-L-A-R (14)
[M+H]+Calc. 1683.7
[M+H]+Exp. 1683.7
T1 [1–14]
MH+
175.034
1021.221
1683.697
1511.676
112.022
86.033
141.044
246.055
332.965
417.967
472.133
559.188
662.965
719.150
790.210
861.205
965.054
1077.234
1136.259
1264.382
1351.256
1592.633
1645.129
200
400
600
800
1000
1200
1400
1600
m/z

Figure S3-2

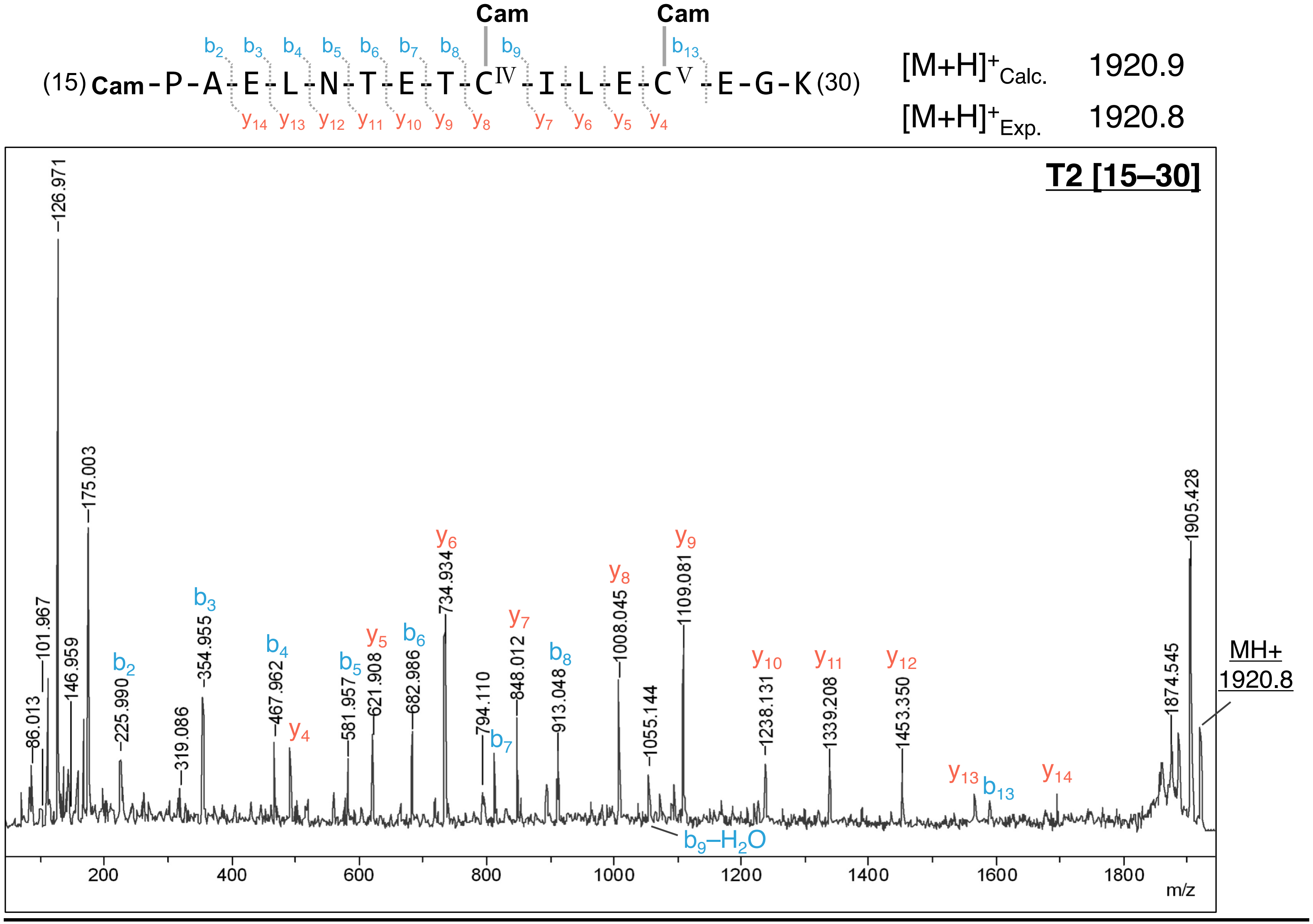

Cam
Cam
b2 b3 b4 b5 b6 b7 b8 b9 b13
(15) Cam-P-A-E-L-N-T-E-T-C IV -I-L-E-C V -E-G-K (30)
y14 y13 y12 y11 y10 y9 y8 y7 y6 y5 y4
[M+H]+ Calc. 1920.9
[M+H]+ Exp. 1920.8
T2 [15–30]
126.971
175.003
86.013
101.967
146.959
225.990
319.086
354.955
467.962
581.957
621.908
682.986
734.934
794.110
848.012
913.048
1008.045
1055.144
1109.081
1238.131
1339.208
1453.350
1874.545
1905.428
MH+
1920.8
b9–H2O
200 400 600 800 1000 1200 1400 1600 1800
m/z

Figure S3-3

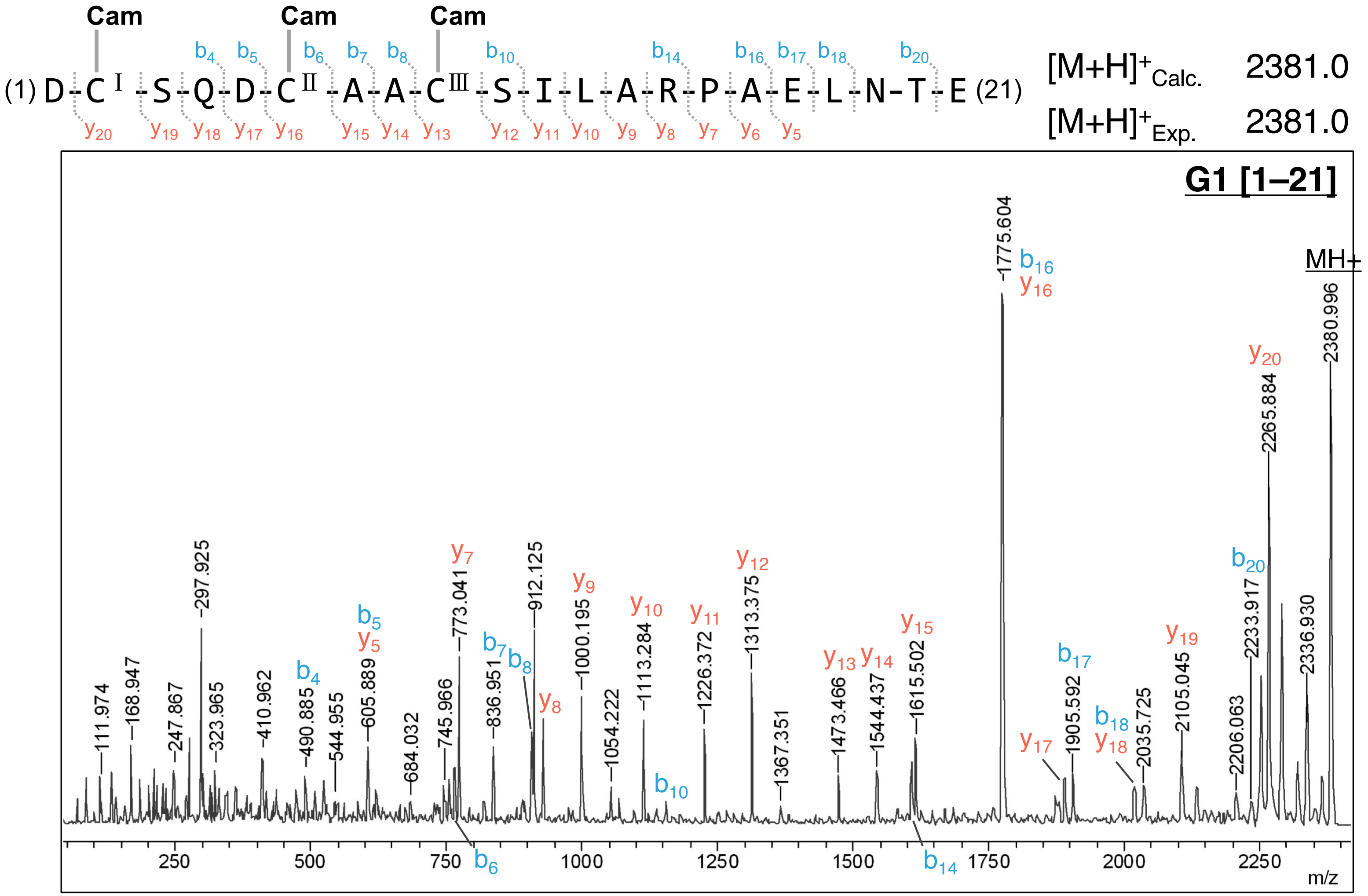
Cam
Cam
Cam
(1) D-C I -S-Q-D-C II -A-A-C III -S-I-L-A-R-P-A-E-L-N-T-E (21)
[M+H]+Calc. 2381.0
[M+H]+Exp. 2381.0
G1 [1–21]
MH+
1775.604
b16 y16
2380.996
y20
2265.884
b20
2233.917
2336.930
y19
2105.045
2206.063
b17
1905.592
y17
b18 y18
2035.725
y15
1615.502
b14
y13 y14
1473.466
1544.437
1367.351
y12
1313.375
y11
1226.372
y10
1113.284
b10
y9
1000.195
1054.222
912.125
b7 b8
y8
836.951
y7
773.041
745.966
b6
684.032
b5 y5
605.889
b4
490.885
544.955
410.962
297.925
323.965
247.867
168.947
111.974
250
500
750
1000
1250
1500
1750
2000
2250
m/z

Figure S3-4

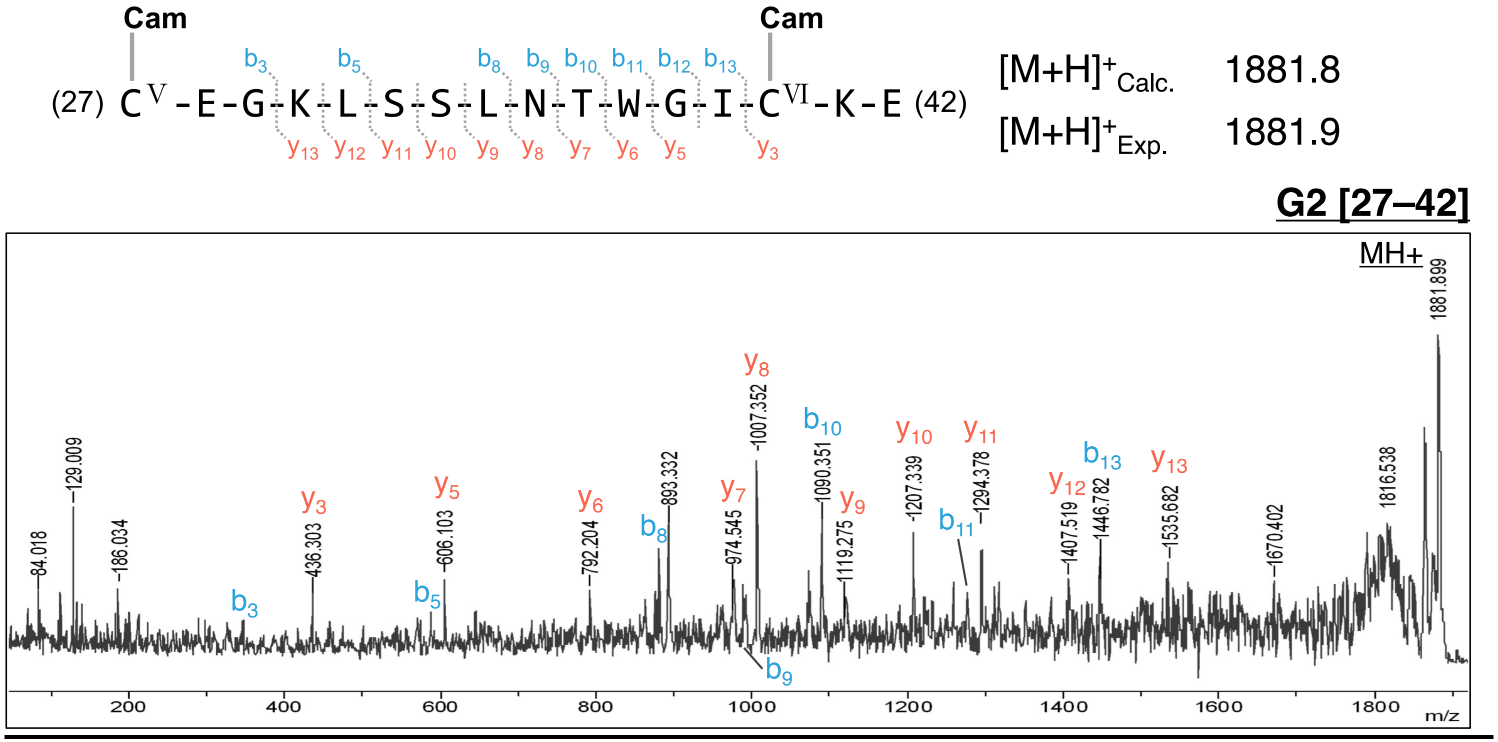

Cam
Cam
(27) C V -E-G-K-L-S-S-L-N-T-W-G-I-C VI -K-E (42)
b3 b5 b8 b9 b10 b11 b12 b13
y13 y12 y11 y10 y9 y8 y7 y6 y5 y3
[M+H]+Calc. 1881.8
[M+H]+Exp. 1881.9
G2 [27–42]
MH+
1881.899
84.018
129.009
186.034
436.303
606.103
792.204
893.332
974.545
1007.352
1090.351
1119.275
1207.339
1294.378
1407.519
1446.782
1535.682
1670.402
1816.538
b3 y3 b5 y5 y6 b8 y7 y8 b9 b10 y9 y10 b11 y11 y12 b13 y13
200 400 600 800 1000 1200 1400 1600 1800
m/z

Figure S3-5

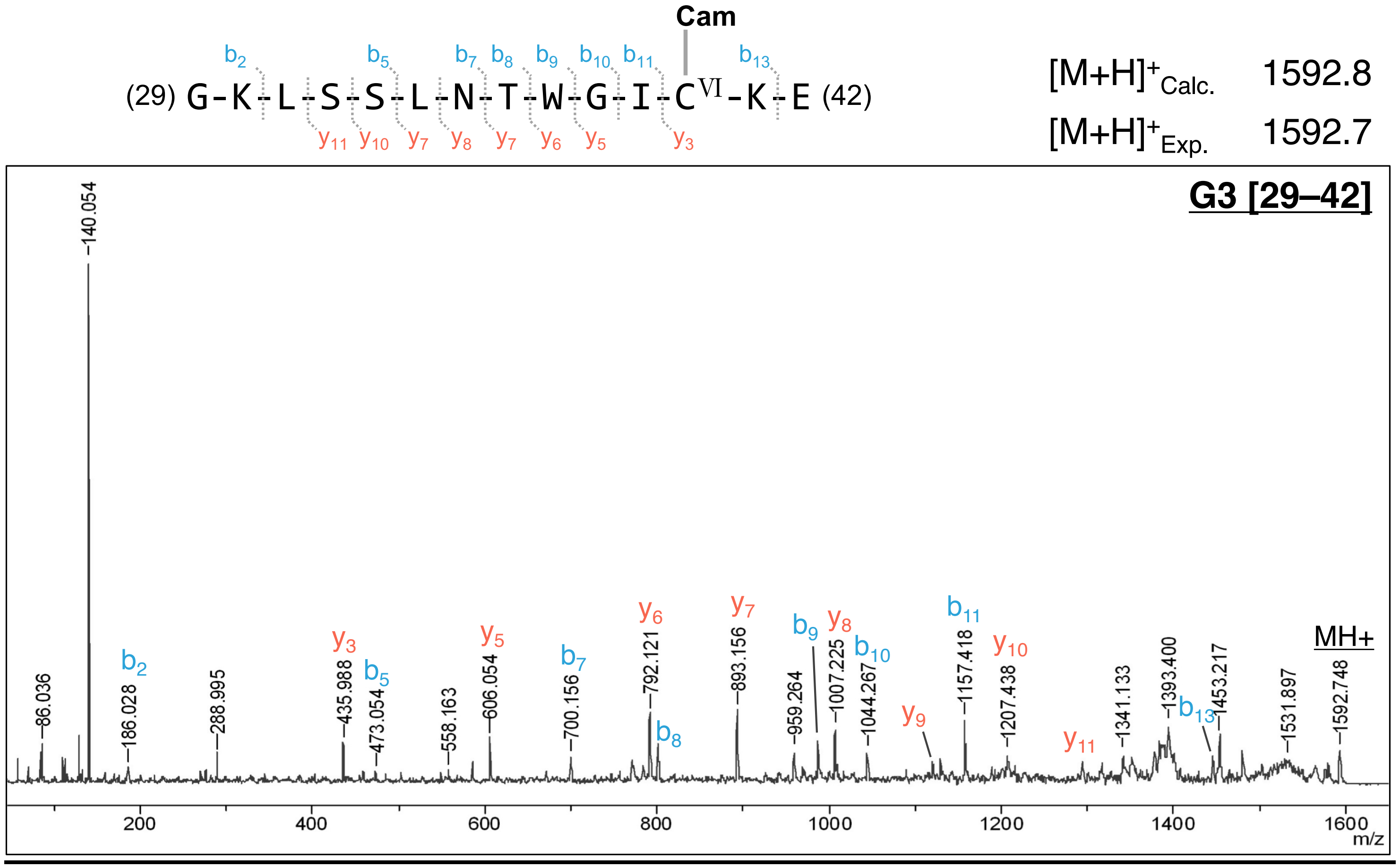
Cam
(29) G-K-L-S-S-L-N-T-W-G-I-C^VI-K-E (42)
b2 b5 b7 b8 b9 b10 b11 b13
y11 y10 y7 y8 y7 y6 y5 y3
[M+H]+Calc. 1592.8
[M+H]+Exp. 1592.7
G3 [29–42]
140.054
86.036
186.028
288.995
435.988
473.054
558.163
606.054
700.156
792.121
893.156
959.264
1007.225
1044.267
1157.418
1207.438
1341.133
1393.400
1453.217
1531.897
1592.748
MH+
m/z

Figure S3-6

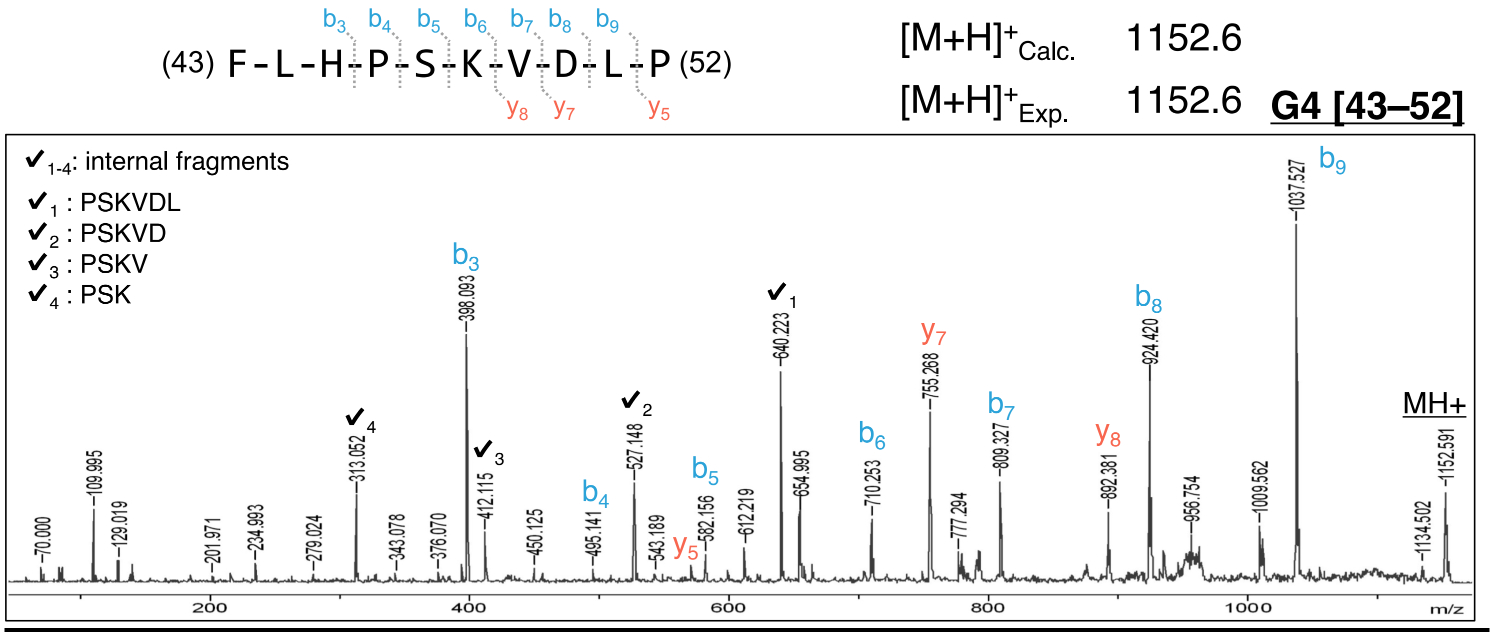

Figure S3. MALDI MS/MS analysis of digested peptides from natural BPP2. For detail, see Fig. S2.

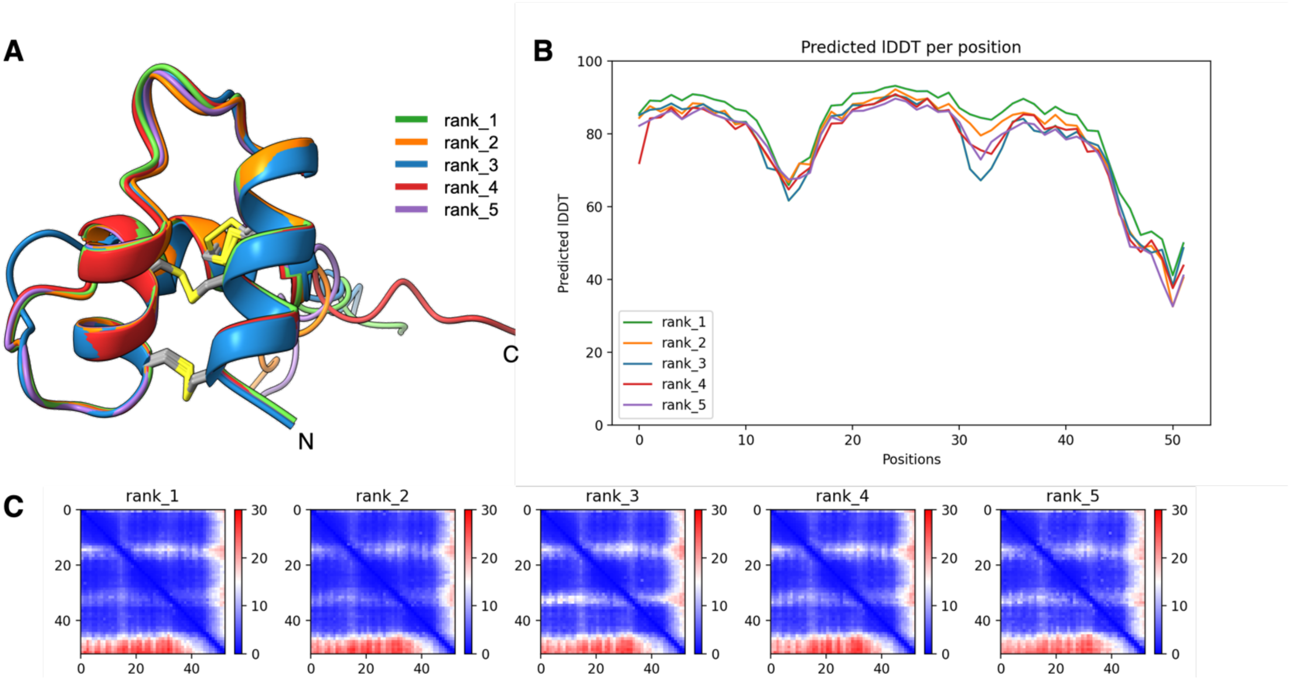

Figure S4. Structure prediction of BPP2 using ColabFold (AlphaFold2 using MMseqs2). **A**: Alignment of five top predicted model structures of BPP2. The pLDDT scores for each model are as follows: rank_1, 81.5; rank_2, 78.3; rank_3, 77.0; rank_4, 76.7; rank_5, 76.5. **B**: The predicted lDDT per residues of the five models. **C**: The calculated predicted aligned errors (PAE) of the five models.

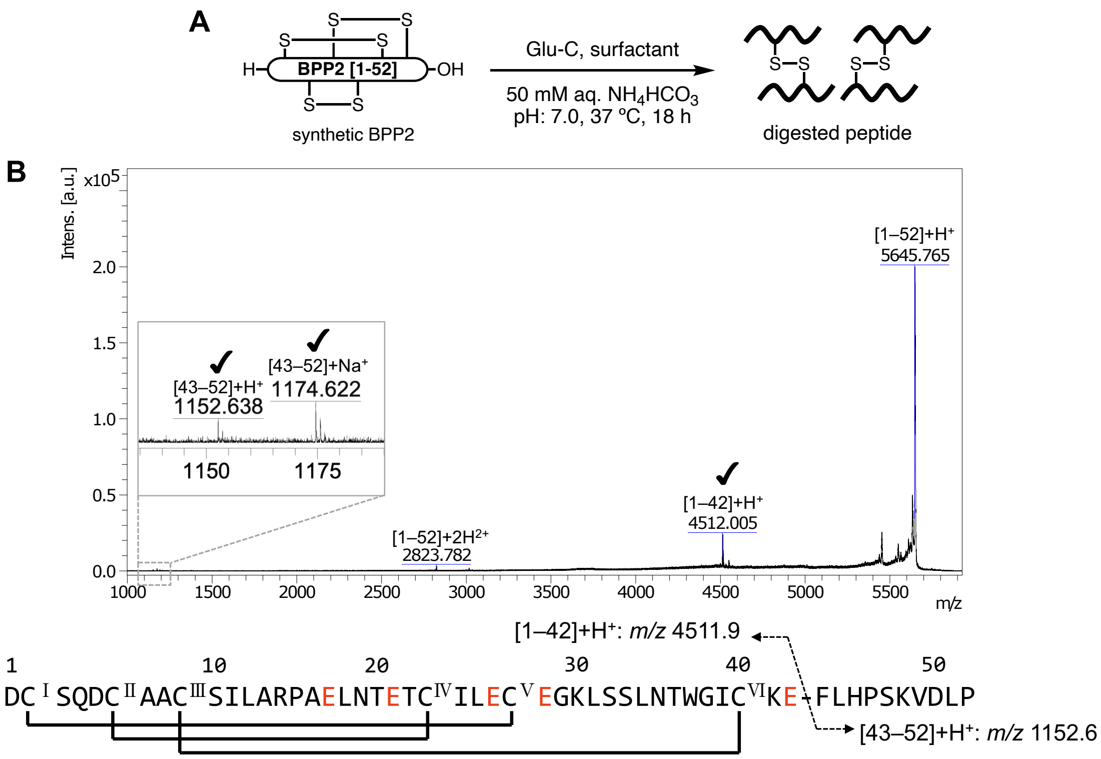

Figure S5. Intact digestion of synthetic BPP2. **A**: Reaction scheme. **B**: MALDI-TOF MS of the mixture of digested peptide fragments. Check marks mean the Glu-C digested peptide fragments.

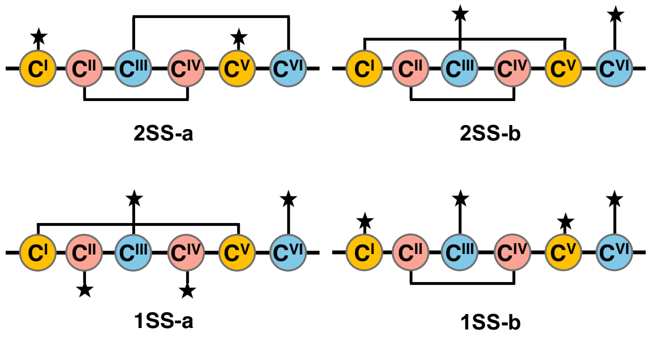

A

Partially *S*-alkylated species	$[M+H]^{+}_{Calc.}$	$[M+H]^{+}_{Exp.}$	Identified fragments
	m/z		
**2SS-a**	2449.0	2449.0	(1) D**C^{I}(NEM)**SQDC^{II}(Cam)AAC^{III}(Cam)SILARPAELNTE (21)
	1949.9	1949.9	(27) **C^{V}(NEM)**EGKLSSLNTWGIC^{VI}(Cam)KE (42)
**2SS-b**	2449.0	2449.0	(1) DC^{I}(Cam)SQDC^{II}(Cam)AA**C^{III}(NEM)**SILARPAELNTE (21)
	1660.8	1660.7	(29) GKLSSLNTWGI**C^{VI}(NEM)**KE (42)
**1SS-a**	2517.1	2517.1	(1) D**C^{I}(Cam)**SQDC^{II}(NEM)AAC^{III}(NEM)SILARPAELNTE (21)
	1949.9	1949.9	(27) **C^{V}(Cam)**EGKLSSLNTWGIC^{VI}(NEM)KE (42)
**1SS-b**	2517.1	2517.1	(1) DC^{I}(NEM)SQD**C^{II}(Cam)**AAC^{III}(NEM)SILARPAELNTE (21)
	2018.0	2018.0	(27) C^{V}(NEM)EGKLSSLNTWGIC^{VI}(NEM)KE (42)

B

Partially *S*-alkylated species	$[M+H]^{+}_{Calc.}$	$[M+H]^{+}_{Exp.}$	Identified fragments
	m/z		
**1SS-a**	2708.1	2708.1	(1) D**C^{I}**SQDC^{II}(NEM)AAC^{III}(NEM)SILARPAELNTE (21)<br>| (27) **C^{V}**E (28)
**1SS-b**	3035.3	3035.3	(1) DC^{I}(NEM)SQD**C^{II}**AAC^{III}(NEM)SILARPAELNTE (21)<br>| (22) T**C^{IV}**ILE (26)

Figure S6. Digested peptide fragments of synthetic BPP2 with partial disulfide bond cleavage. The disulfide bond connectivity was determined based on cysteine residue pairs (bold, understand). Monoisotopic masses are used for calculated and measured *m/z*. **A**: Assigned fragment peptides of **2SS** and **1SS** with Cam alkylation. **B**: Assigned fragment peptides of **1SS** without Cam alkylation.

Figure S7-1

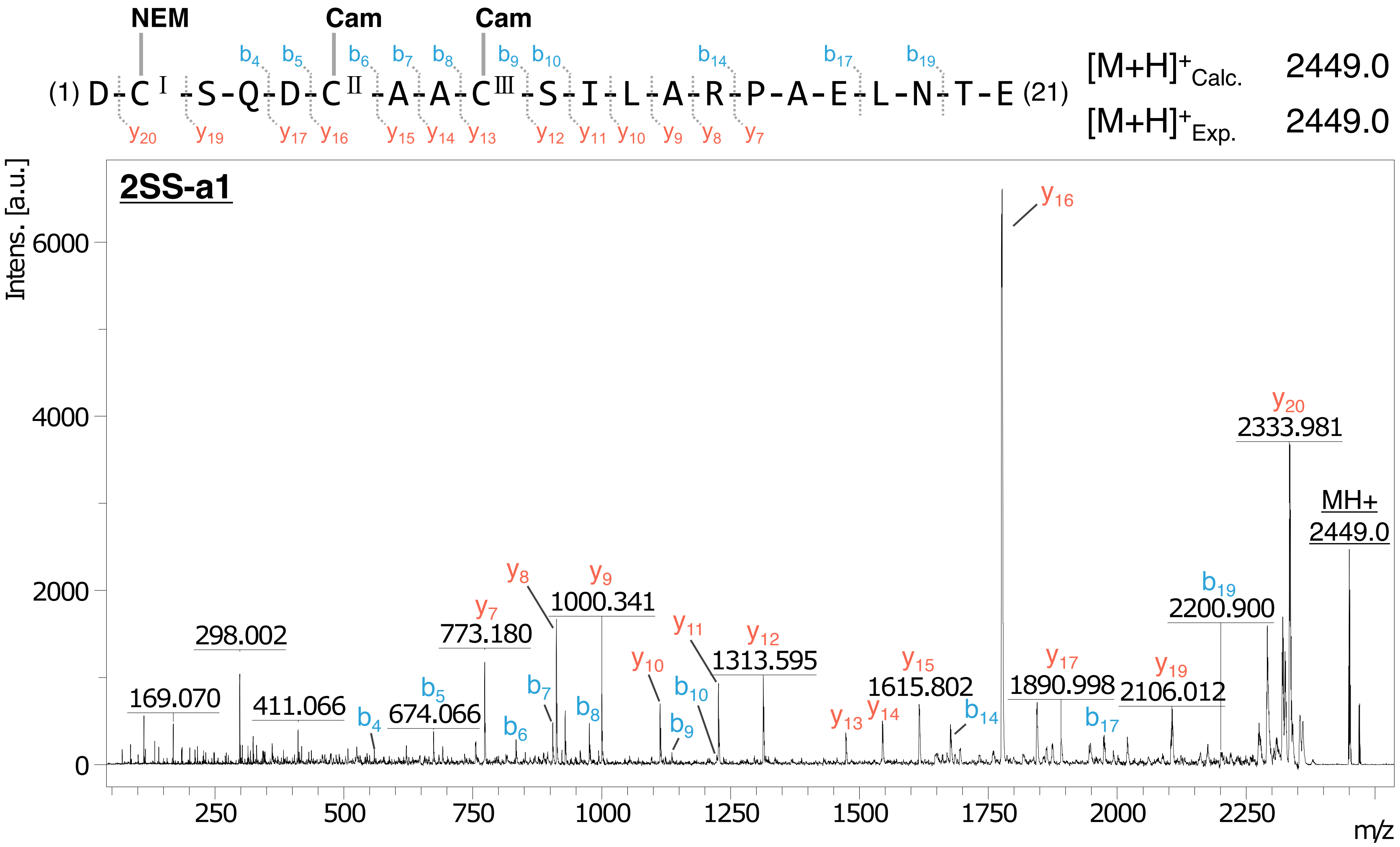
NEM
Cam
Cam
(1) D-C I -S-Q-D-C II -A-A-C III -S-I-L-A-R-P-A-E-L-N-T-E (21)
[M+H]+ Calc. 2449.0
[M+H]+ Exp. 2449.0
2SS-a1
Intens. [a.u.]
m/z
y16
y20
2333.981
MH+
2449.0
b19
2200.900
y8
y9
1000.341
y7
773.180
298.002
169.070
411.066
674.066
y11
1313.595
y12
y15
1615.802
y17
1890.998
y19
2106.012

Figure S7-2

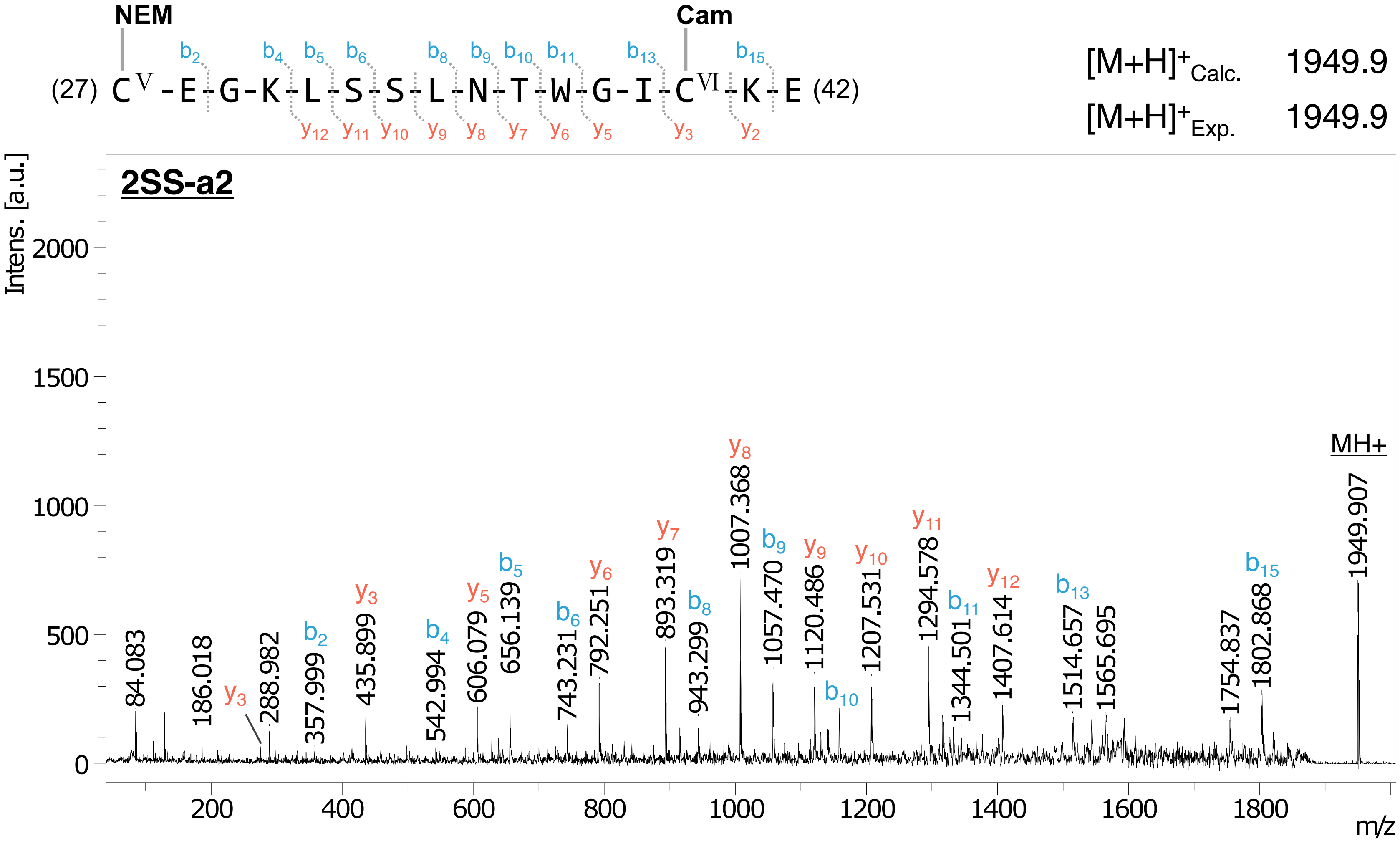
NEM
Cam
(27) C V -E-G-K-L-S-S-L-N-T-W-G-I-C VI -K-E (42)
b2 b4 b5 b6 b8 b9 b10 b11 b13 b15
y12 y11 y10 y9 y8 y7 y6 y5 y3 y2
[M+H]+Calc. 1949.9
[M+H]+Exp. 1949.9
2SS-a2
Intens. [a.u.]
m/z
MH+
1949.907
84.083
186.018
288.982
357.999
435.899
542.994
606.079
656.139
743.231
792.251
893.319
943.299
1007.368
1057.470
1120.486
1207.531
1294.578
1344.501
1407.614
1514.657
1565.695
1754.837
1802.868

Figure S7-3

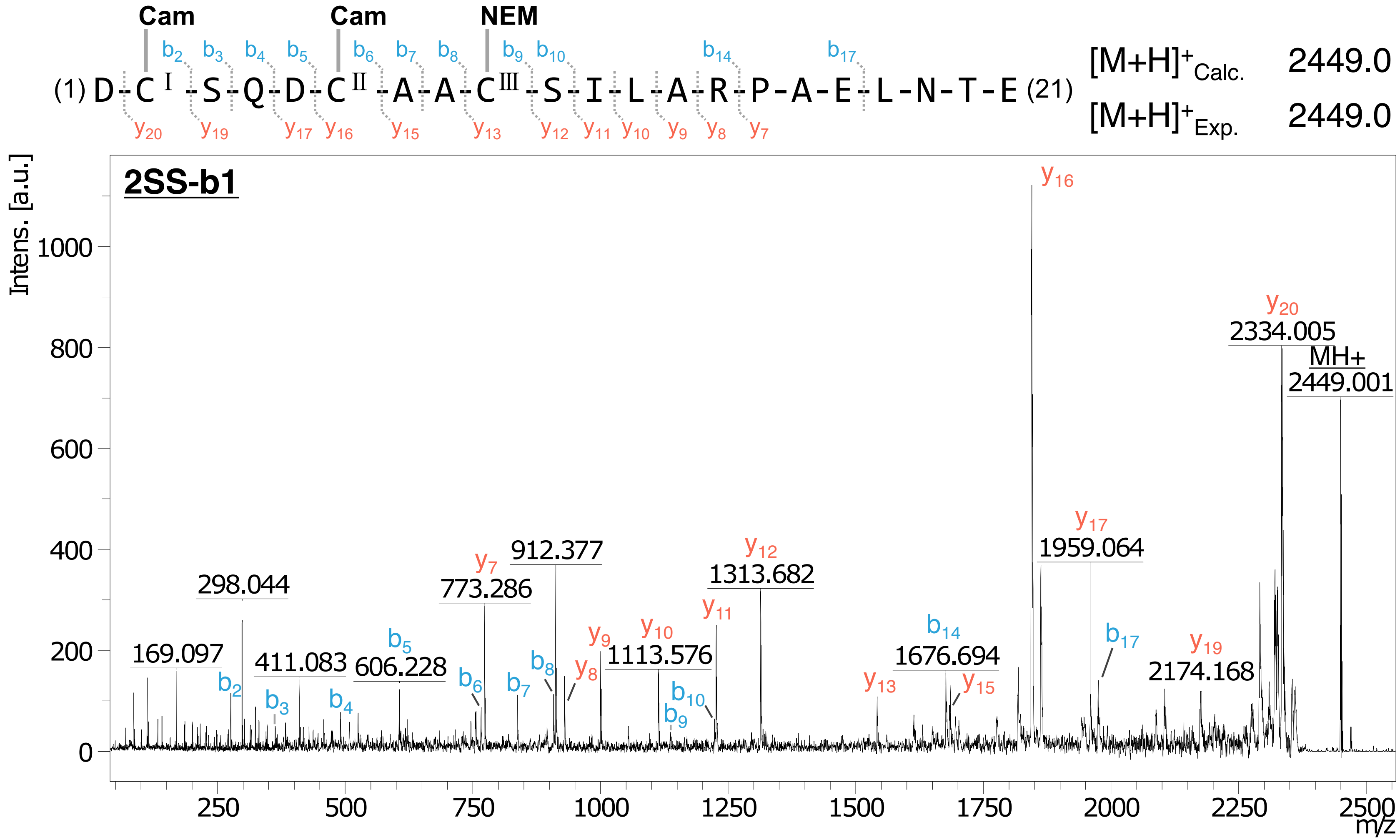

Cam
Cam
NEM
(1) D-C I -S-Q-D-C II -A-A-C III -S-I-L-A-R-P-A-E-L-N-T-E (21)
[M+H]+Calc. 2449.0
[M+H]+Exp. 2449.0
2SS-b1
Intens. [a.u.]
m/z
y16
y20
2334.005
MH+
2449.001
y17
1959.064
912.377
y7
773.286
y12
1313.682
298.044
169.097
411.083
606.228
1113.576
1676.694
2174.168

Figure S7-4

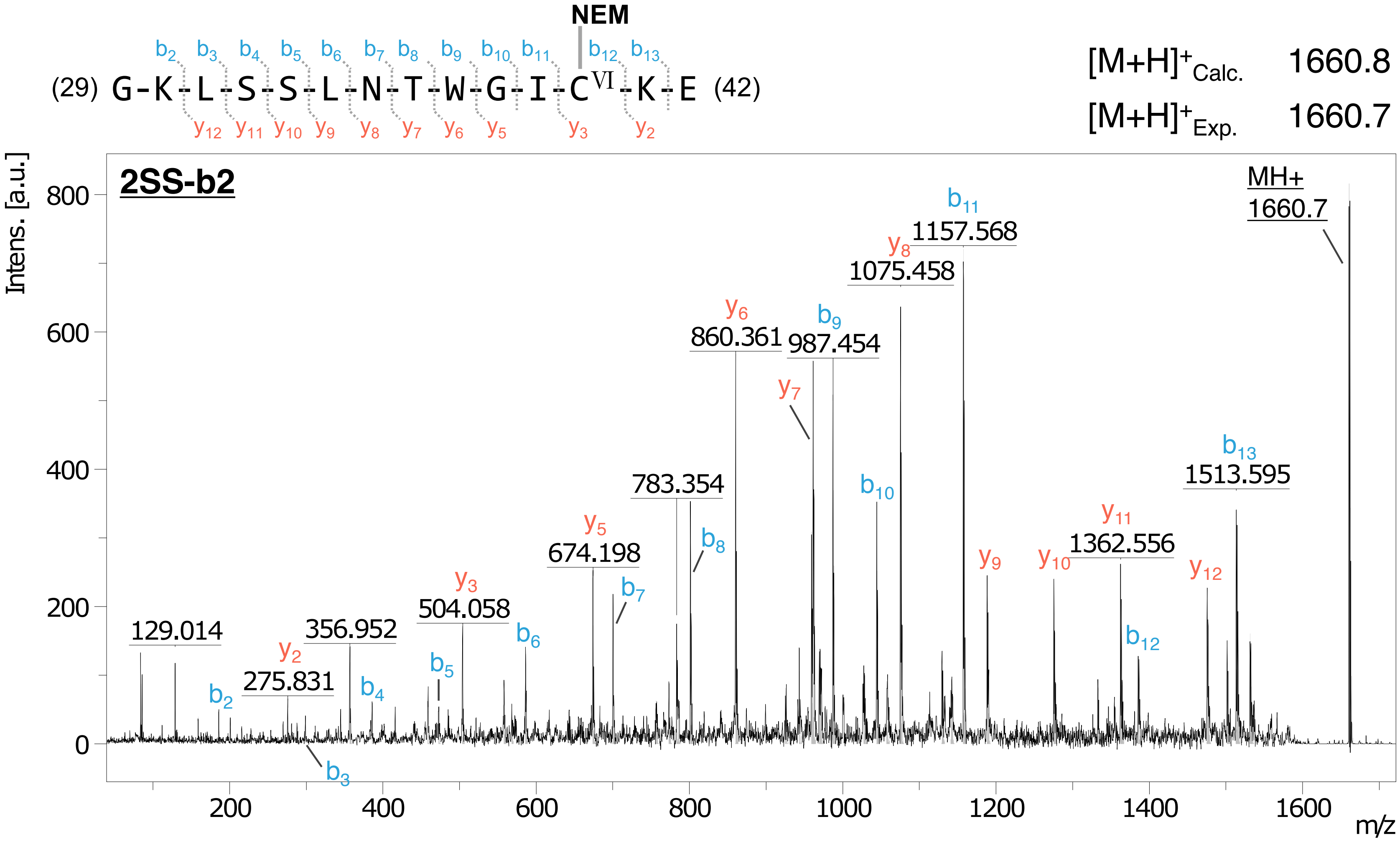
NEM
(29) G-K-L-S-S-L-N-T-W-G-I-C VI -K-E (42)
b2 b3 b4 b5 b6 b7 b8 b9 b10 b11 b12 b13
y12 y11 y10 y9 y8 y7 y6 y5 y3 y2
[M+H]+Calc. 1660.8
[M+H]+Exp. 1660.7
2SS-b2
Intens. [a.u.]
MH+ 1660.7
b11 1157.568
y8 1075.458
y6 860.361
b9 987.454
y7
783.354
b8
b10
b13 1513.595
y11 1362.556
y5 674.198
b7
y9
y10
y12
y3 504.058
b6
b12
129.014
356.952
y2 275.831
b2
b4
b5
b3
m/z

Figure S7-5

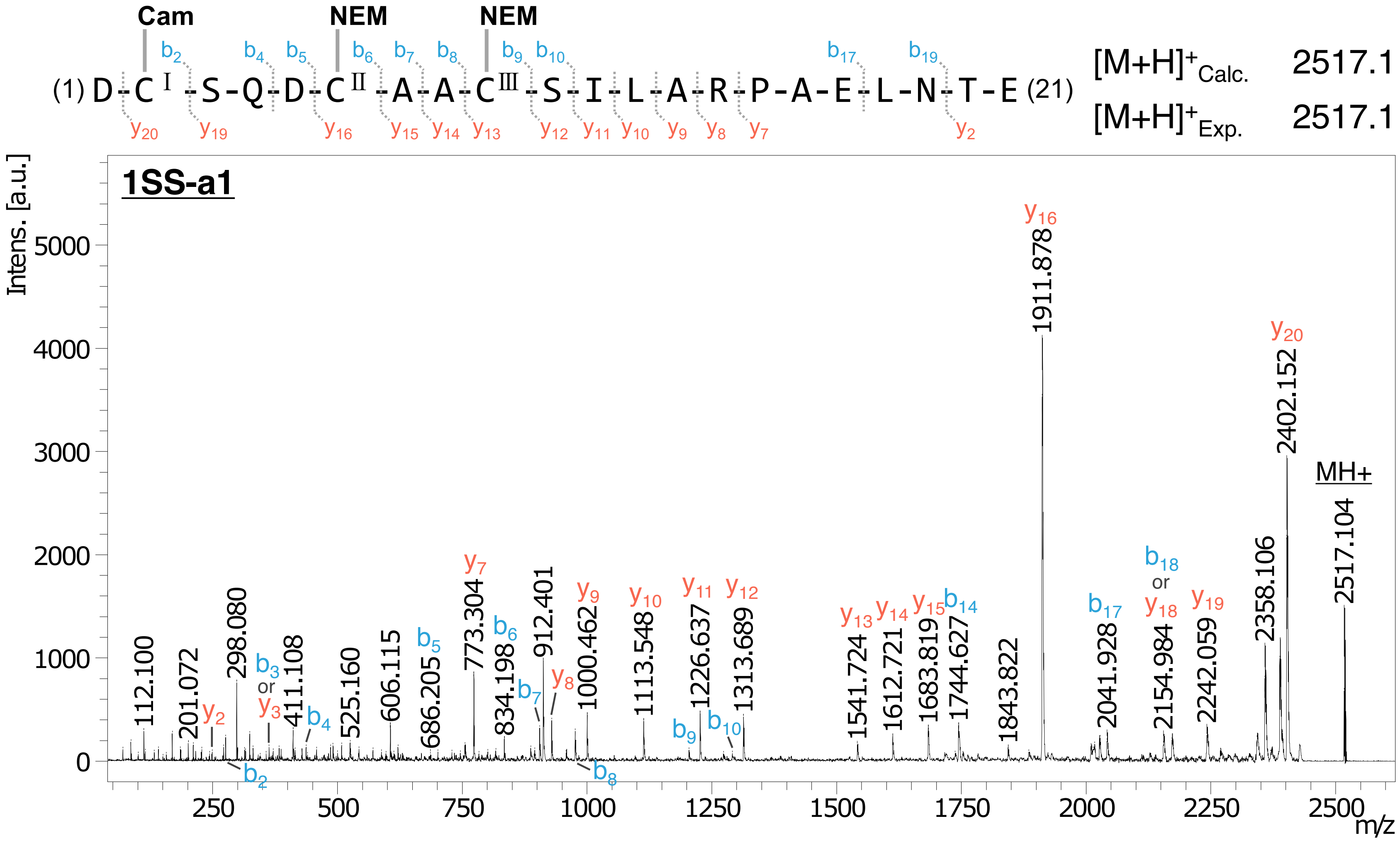

Cam
NEM
NEM
(1) D-C I -S-Q-D-C II -A-A-C III -S-I-L-A-R-P-A-E-L-N-T-E (21)
b2 b4 b5 b6 b7 b8 b9 b10 b17 b19
y20 y19 y16 y15 y14 y13 y12 y11 y10 y9 y8 y7 y2
[M+H]+Calc. 2517.1
[M+H]+Exp. 2517.1
1SS-a1
Intens. [a.u.]
5000
4000
3000
2000
1000
0
y16 1911.878
y20 2402.152
MH+ 2517.104
112.100
201.072
y2
298.080
b3 or y3
411.108
b4
b2
525.160
606.115
b5
686.205
y7 773.304
b6
834.198
b7
912.401
y8
y9 1000.462
b8
y10 1113.548
b9
y11 1226.637
b10
y12 1313.689
y13 1541.724
y14 1612.721
y15 1683.819
b14 1744.627
1843.822
b17 2041.928
b18 or y18 2154.984
y19 2242.059
2358.106
250
500
750
1000
1250
1500
1750
2000
2250
2500
m/z

Figure S7-6

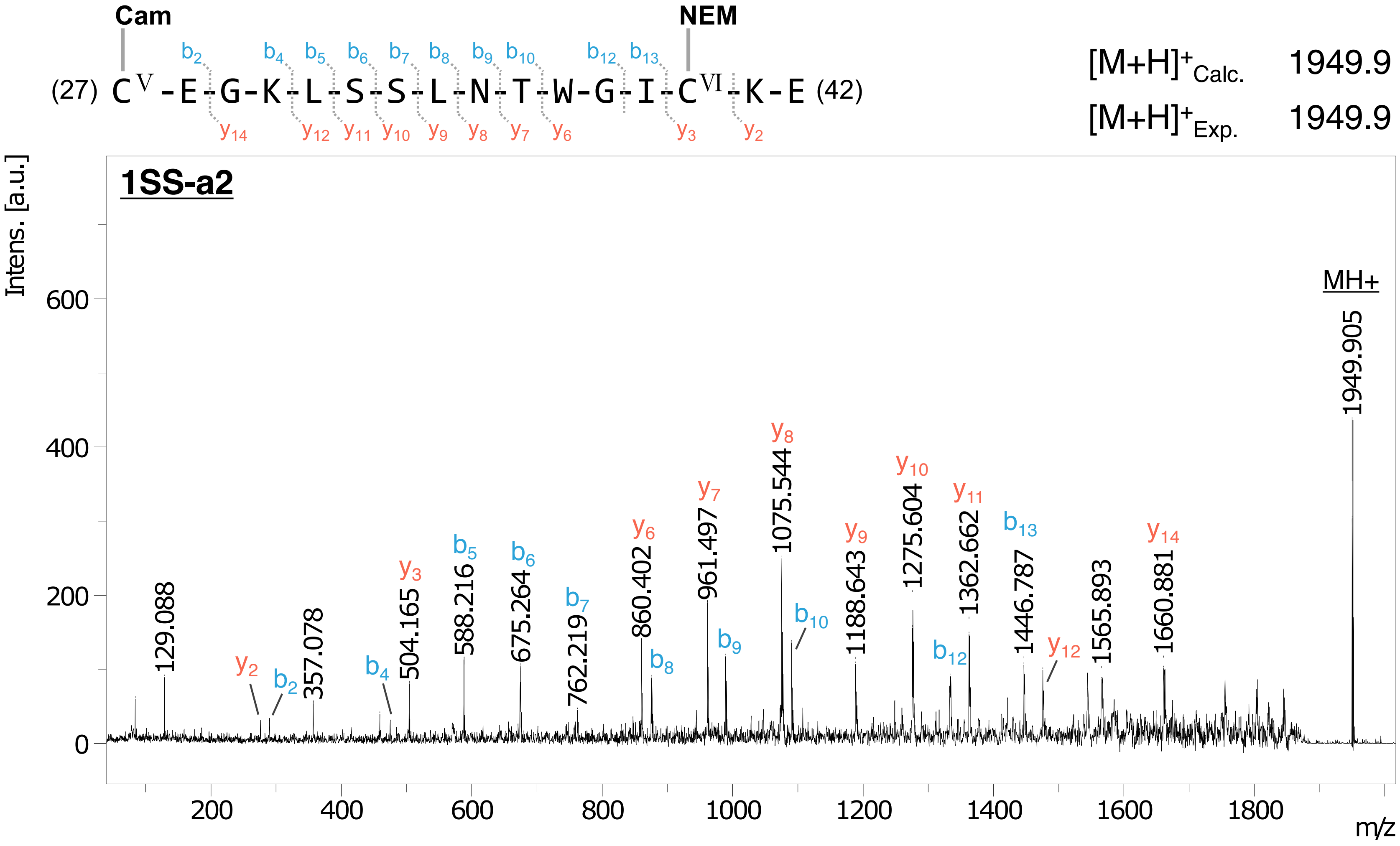

Cam
NEM
(27) C V -E-G-K-L-S-S-L-N-T-W-G-I-C VI -K-E (42)
b2 b4 b5 b6 b7 b8 b9 b10 b12 b13
y14 y12 y11 y10 y9 y8 y7 y6 y3 y2
[M+H]+Calc. 1949.9
[M+H]+Exp. 1949.9
1SS-a2
Intens. [a.u.]
MH+
1949.905
1075.544
1275.604
1362.662
1446.787
1565.893
1660.881
961.497
860.402
1188.643
762.219
675.264
588.216
504.165
357.078
129.088
m/z

Figure S7-7

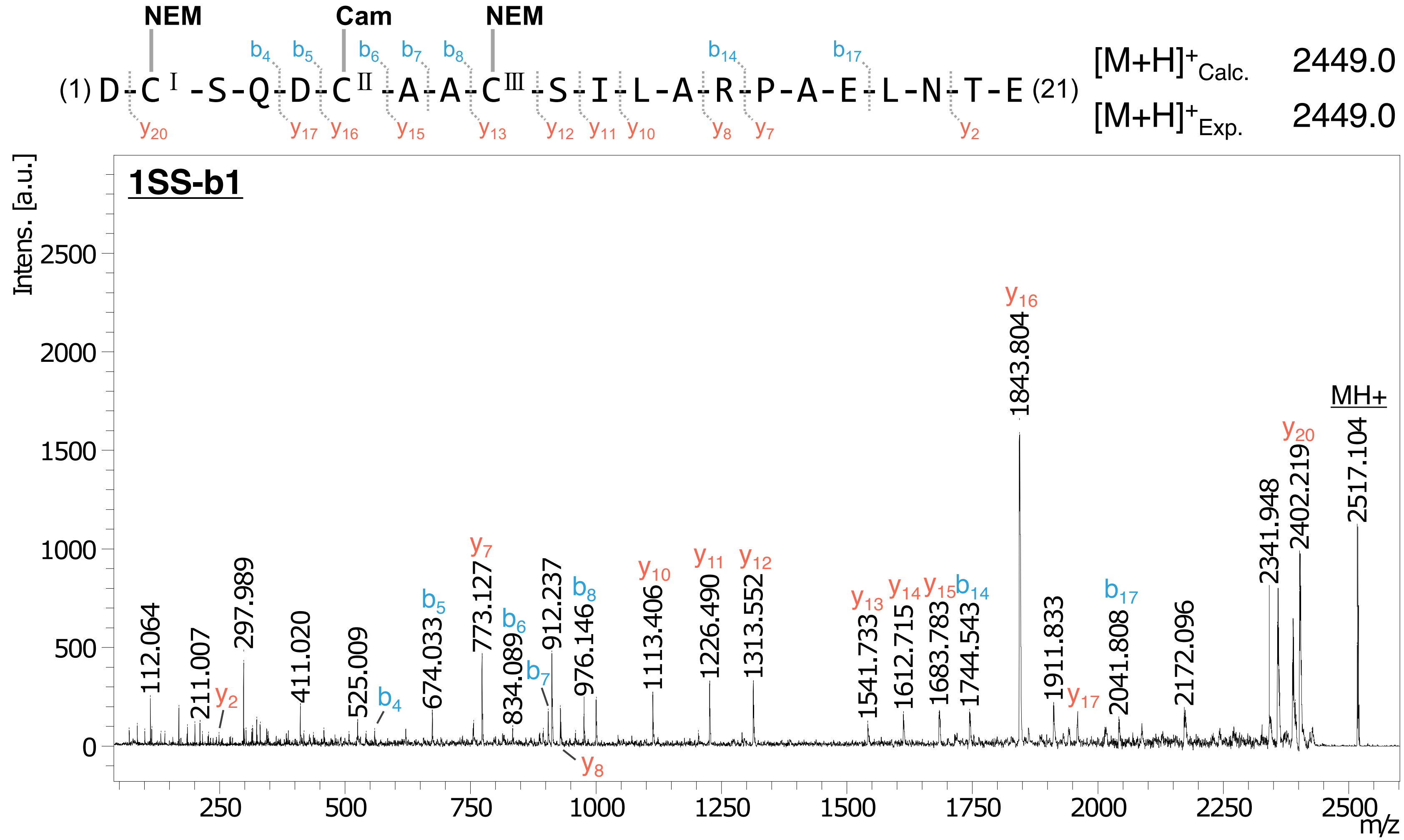
NEM
Cam
NEM
(1) D-C I -S-Q-D-C II -A-A-C III -S-I-L-A-R-P-A-E-L-N-T-E (21)
b4 b5 b6 b7 b8 b14 b17
y20 y17 y16 y15 y13 y12 y11 y10 y8 y7 y2
[M+H]+Calc. 2449.0
[M+H]+Exp. 2449.0
1SS-b1
Intens. [a.u.]
m/z
MH+
112.064
211.007
297.989
411.020
525.009
674.033
773.127
834.089
912.237
976.146
1113.406
1226.490
1313.552
1541.733
1612.715
1683.783
1744.543
1843.804
1911.833
2041.808
2172.096
2341.948
2402.219
2517.104

Figure S7-8

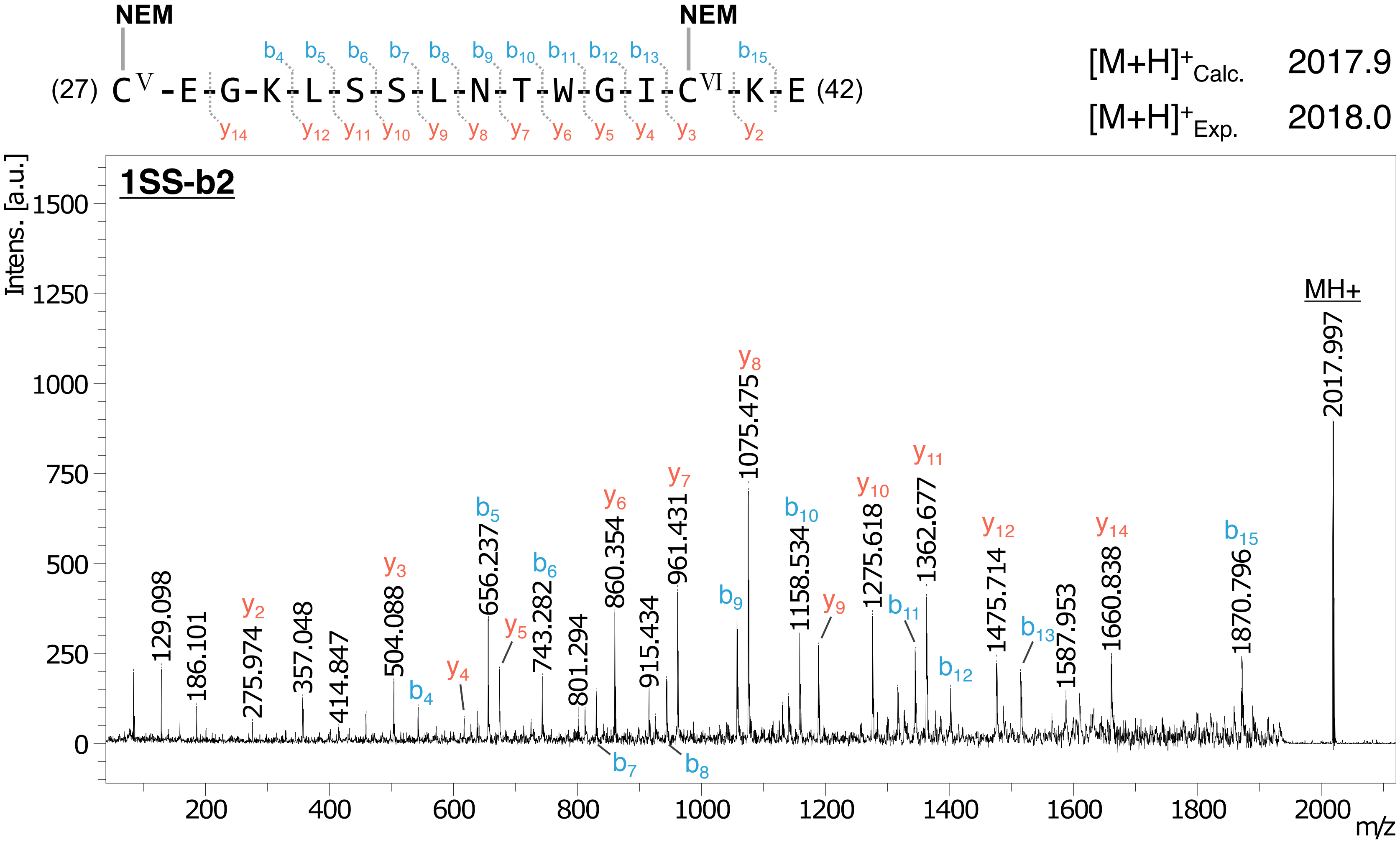

NEM
NEM
(27) C V -E-G-K-L-S-S-L-N-T-W-G-I-C VI -K-E (42)
b4 b5 b6 b7 b8 b9 b10 b11 b12 b13 b15
y14 y12 y11 y10 y9 y8 y7 y6 y5 y4 y3 y2
[M+H]+Calc. 2017.9
[M+H]+Exp. 2018.0
1SS-b2
Intens. [a.u.]
MH+
2017.997
1075.475
1362.677
1275.618
1158.534
961.431
860.354
656.237
1475.714
1660.838
1870.796
1587.953
743.282
915.434
801.294
504.088
129.098
186.101
275.974
357.048
414.847
m/z

Figure S7-9

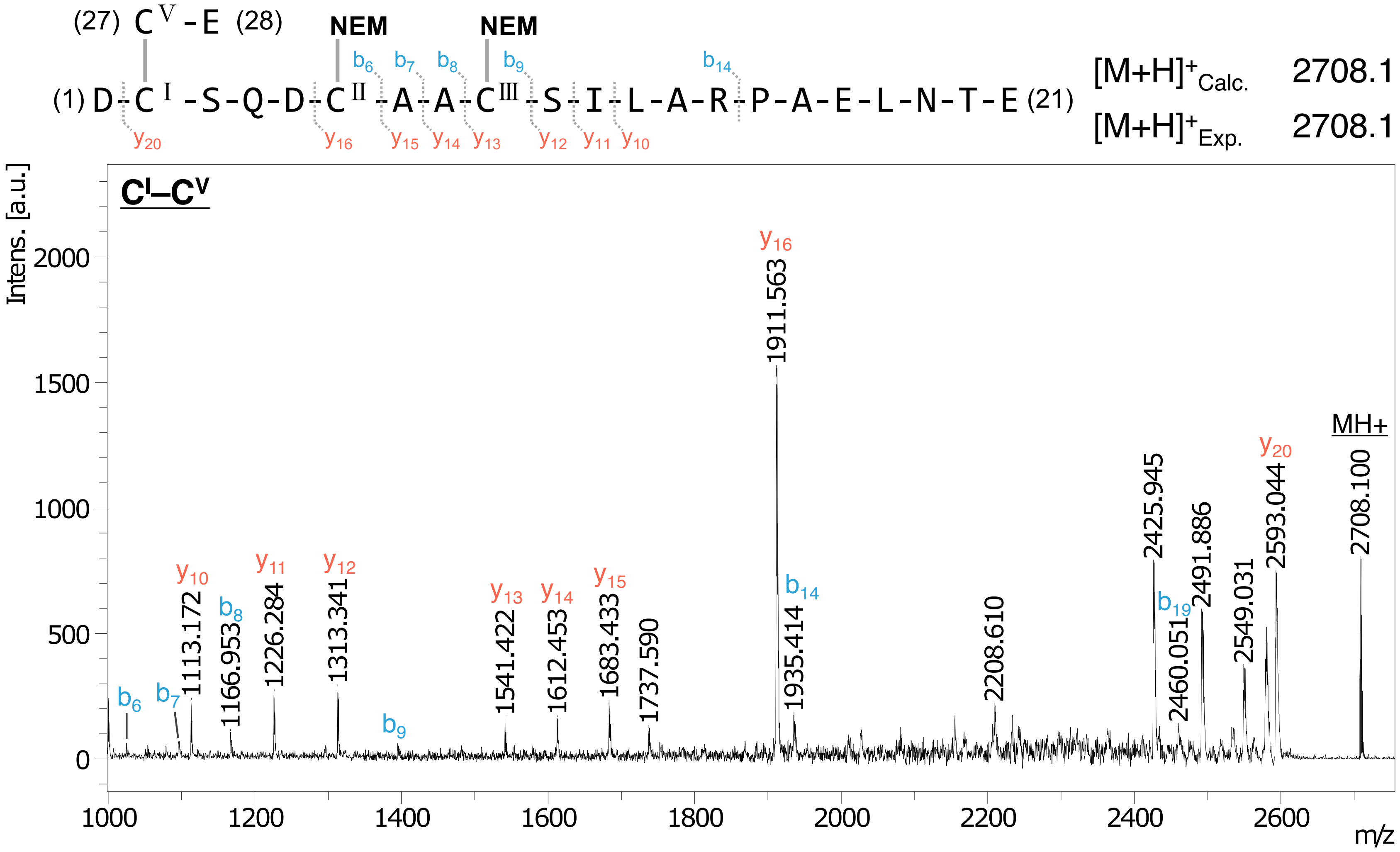

(27) C V - E (28)
NEM
NEM
(1) D-C I -S-Q-D-C II -A-A-C III -S-I-L-A-R-P-A-E-L-N-T-E (21)
b6 b7 b8 b9 b14
y20 y16 y15 y14 y13 y12 y11 y10
[M+H]+Calc. 2708.1
[M+H]+Exp. 2708.1
CI–CV
Intens. [a.u.]
m/z
MH+
1911.563
2708.100
2593.044
2425.945
2491.886
2549.031
2460.051
2208.610
1935.414
1737.590
1683.433
1612.453
1541.422
1313.341
1226.284
1166.953
1113.172

Figure S7-10

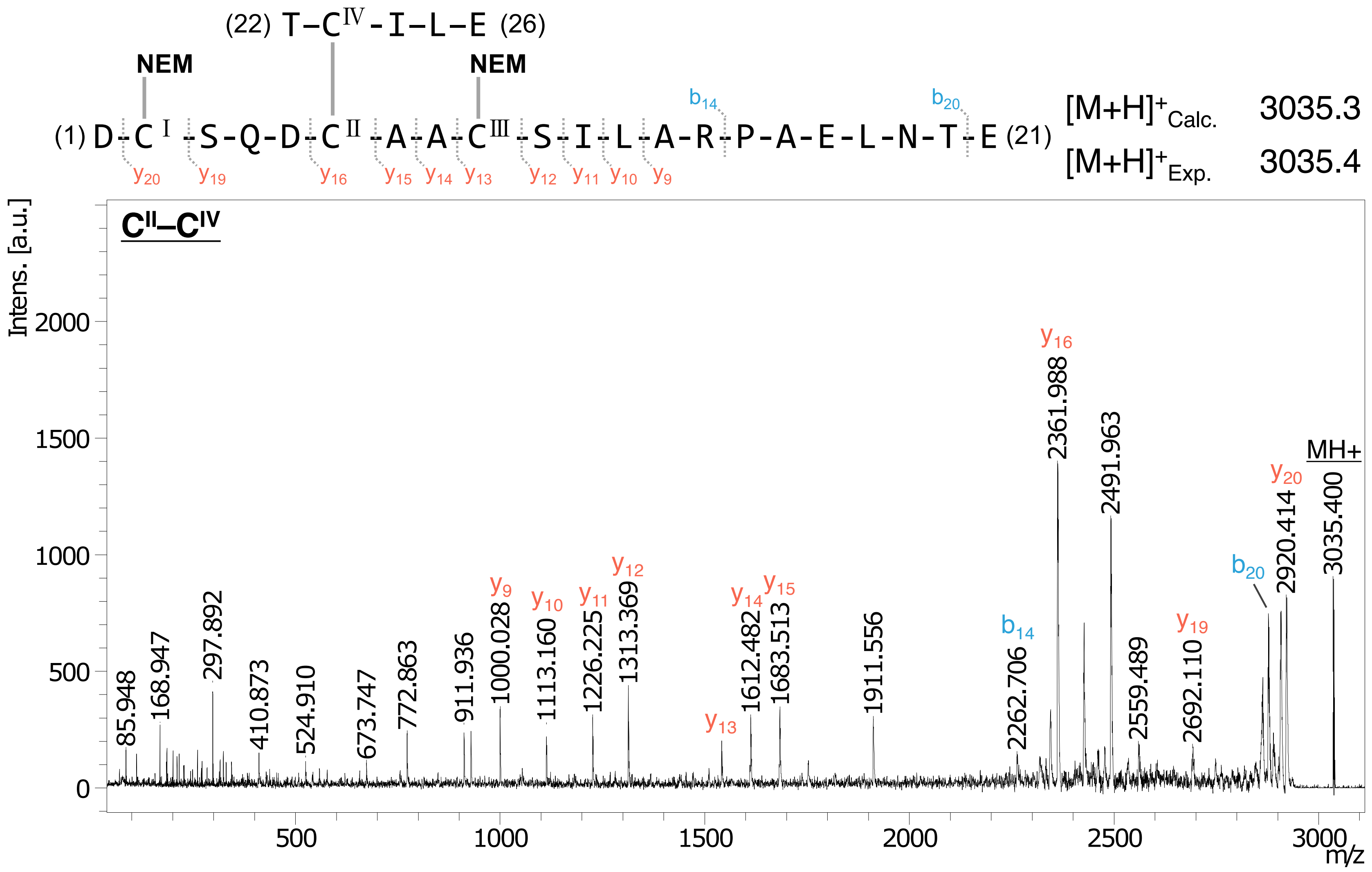

Figure S7. MALDI MS/MS analysis of digested peptides from synthetic BPP2 with partial disulfide bond cleavage. For detail, see Figure S6.

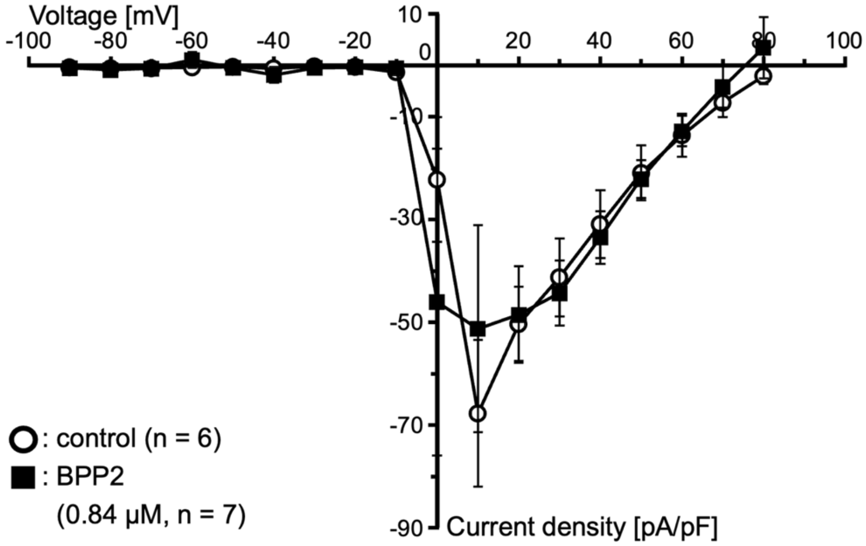

Figure S8. Voltage-gated Ca^{2+} currents measured by whole-cell patch-clamp experiments using HEK293T cells expressing $hCa_v2.2$. The current density–voltage relationships were recorded for cells treated with (■) or without (○) synthetic BPP2. These are shown as data ± S.E.M.

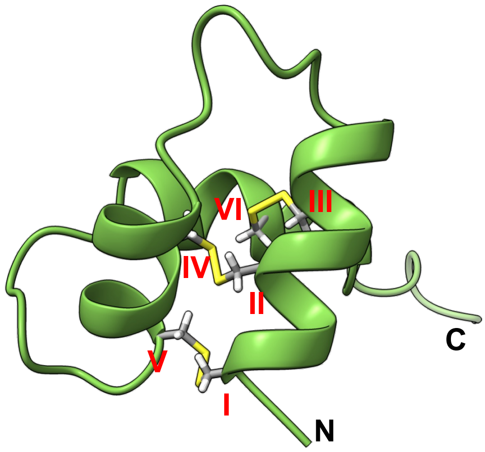

	PDBMD2CD	K2D3	BestSel
Predicted	55.7	54.9	48.9
Measured	–	55.6	45.2

(% helicity)

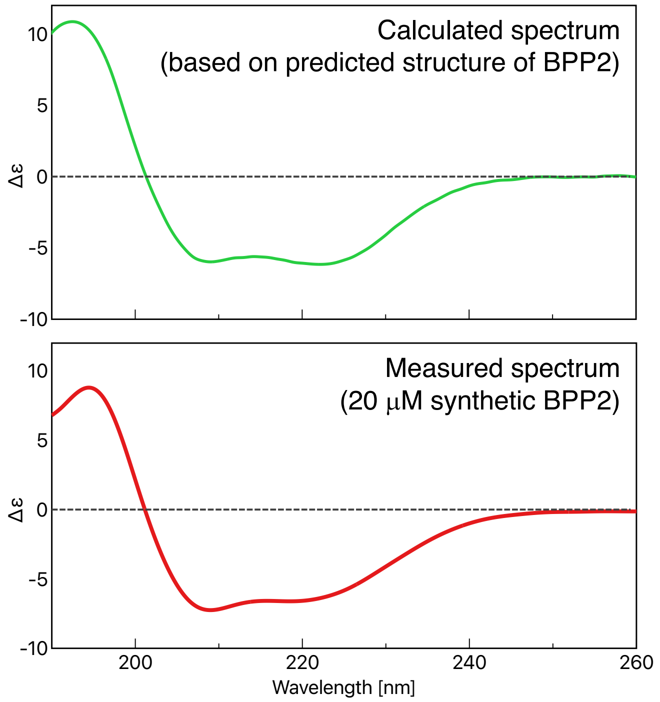

Figure S9. CD analysis of BPP2. Calculated (right top) by PDBMD2CD (81) and measured (right bottom) spectra are shown. Two negative bands at 208 and 222 nm and a positive band at 195 nm were observed in both spectra. Helical contains of BPP2 are shown in the left bottom, which were estimated by PDBMD2CD from the proposed stereostructure (left top), and by K2D3 (82) and BeStSel (83) from the CD spectra.

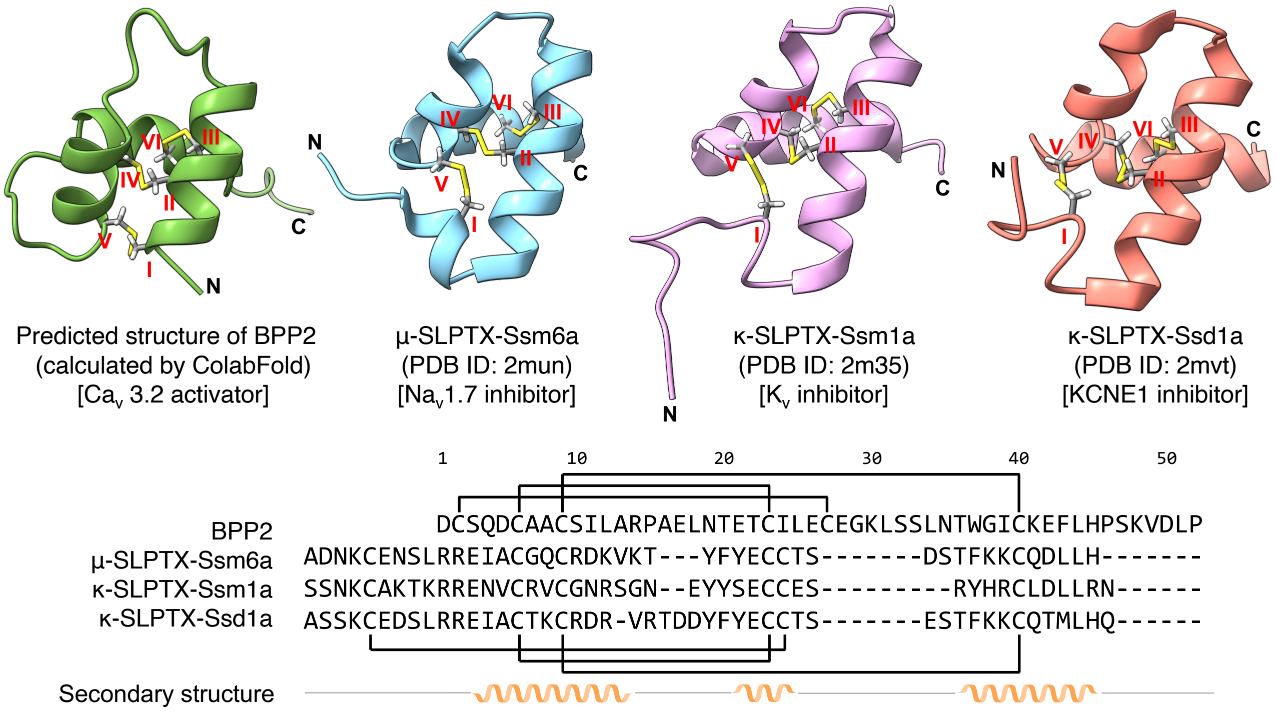

Figure S10. Structural homology search of BPPs using the Dali server (84). Three peptides (PDB ID: 2mun, 2m35, 2mvt) obtained by the Dali search are shown with ribbon representations, along with the predicted structure of BPP2 shown in Fig. 2C. Results of structural homology searches are shown in bottom, in which three α-helix structures and three disulfide bonds are indicated.

Table S1. Paralytic activity of BPP2 against mealworms.

Dose (μg/g body weight)	total numbers	paralyzed numbers
0 [a]	3	0
0.056	3	0
0.56	3	0
5.6	3	3

[a] PBS (100 μL per 1.0 g mealworm bodyweight) was injected as a control.
